# On Globalized Traces for the Poisson Sigma Model

**DOI:** 10.1007/s00220-022-04371-4

**Published:** 2022-04-27

**Authors:** Nima Moshayedi

**Affiliations:** 1 Institut für Mathematik, Universität Zürich, Winterthurerstrasse 190, 8057 Zürich, Switzerland; 2 UC Berkeley, Berkeley, CA USA

## Abstract

A globalized version of a trace formula for the Poisson Sigma Model on the disk is presented by using its formal global picture in the setting of the Batalin–Vilkovisky formalism. This global construction includes the concept of zero modes. Moreover, for the symplectic case of the Poisson Sigma Model with cotangent target, the globalized trace reduces to a symplectic construction which was presented by Grady, Li and Li for 1-dimensional Chern–Simons theory (topological quantum mechanics). In addition, the connection between this formula and the Nest–Tsygan theorem and the Tamarkin–Tsygan theorem is explained.

## Introduction

In [36] Kontsevich showed that the differential graded Lie algebra (DGLA) of multidifferential operators on a manifold *M* is $L_\infty$-quasi-isomorphic to the DGLA of multivector fields on *M*. This is known as the formality theorem. The construction of Kontsevich’s star product in deformation quantization is given by the special case of the formality theorem for bivector fields and bidifferential operators. In [11] it was shown that this star product can be written as a perturbative expansion of a path integral given by the Poisson Sigma Model [34, 38]. In [42] Tsygan formulated a formality conjecture for cyclic chains (which was motivated as a chain version of the Connes–Flato–Sternheimer cyclic cohomology construction [23]), which was partially proven by Shoikhet [40], Dolgushev [24] and Willwacher [46]. In [47] Willwacher and Calaque have proven the cyclic formality conjecture of Kontsevich, which was the formulation for cyclic cochains.

A global geometrical picture of the star product coming from the Weyl quantization approach for symplectic manifolds, i.e. for a constant Poisson structure, was given by Fedosov in [26]. There one chooses a(n) (always existing) symplectic connection and its corresponding exponential map. This construction can be generalized to the local picture of Kontsevich’s star product to produce a global version on any Poisson manifold [18, 19], where one uses notions of formal geometry [9, 29]. The symplectic connection (lifted to the Weyl bundle) can be replaced by the (deformed) Grothendieck connection which is constructed by using any (formal) exponential map (see also [17]). A globalized picture in the field theoretic approach using the Poisson Sigma Model in the Batalin–Vilkovisky (BV) formalism [4–6] was given in [8] for closed worldsheet manifolds and in [21] for manifolds with boundary using the BV-BFV formalism [12–15]. Here BFV stands for Batalin–Fradkin–Vilkovisky, which is the Hamiltonian approach of the BV formalism developed in [2, 3].

An important object to study for closed^1^ star products [23] are trace maps. In [37] Nest and Tsygan showed an algebraic version of the Atiyah–Singer index theorem, where they made the link to a trace map with respect to the underlying star product and computed the index as the trace of the constant function 1 (see also [27] for Fedosov’s construction). This construction is given for symplectic manifolds together with the globalization construction of the Moyal product. For a general Poisson manifold with Kontsevich’s star product, Cattaneo and Felder constructed a trace map in terms of local field theoretic constructions using the Poisson Sigma Model on the disk for negative cyclic chains [16] in the presence of residual fields (a.k.a “slow” fields,“low energy” fields).

We will extend this construction to a global one by using a formal global version of the Poisson Sigma Model. This construction in fact combines Fedosov’s globalization construction with field theoretic concepts on Poisson manifolds and the BV formulation. We also give the connection of the obtained globalized trace to the Tamarkin–Tsygan theorem, which can be seen as a cyclic equivariant extension of the Nest–Tsygan theorem for Poisson manifolds using formally extended Poisson structures. The connection can be understood by field theoretic concepts by looking at the Feynman graph expansion for the obtained trace formula, which geometrically gives rise to a deformed version of the Grothendieck connection and its curvature. We would also like to refer to the work of Dolgushev and Rubtsov [25], who proposed a version of an algebraic index theorem for Poisson manifolds using a *trace density map*.

In [33] a global equivariant trace formula for symplectic manifolds was constructed by using a Fedosov connection and solutions to the Fedosov equation. The field theoretic construction was given by the effective theory of topological quantum mechanics on the circle $S^1$. We show that our trace formula reduces to this trace formula if we consider the Poisson sigma model with cotangent target. To show this, we use the fact (Proposition 4.2) that the vertices of our graphs in the expansion which arise from the Grothendieck connection are linear in the fiber coordinates if the underlying manifold is a cotangent bundle.

### Main results

We prove that the map ${{\,\mathrm{Tr}\,}}^\mathbb {D}$, constructed out of a globalization construction for the Poisson Sigma Model on the disk $\mathbb {D}$, coincides with the map ${{\,\mathrm{Tr}\,}}^\mathcal {V}$, constructed out of curvature terms of Kontsevich’s formality map and negative cyclic chains, which is the statement of Proposition 6.7. Then we prove that the map ${{\,\mathrm{Tr}\,}}^\mathcal {V}$ is indeed a trace on $(C_c^\infty (M)[\![\hbar ]\!],\star )$, which is the statement of Theorem 8.1. Moreover, we show how the map ${{\,\mathrm{Tr}\,}}^\mathcal {V}$ is actually related to the Tamarkin–Tsygan theorem for the Poisson case and to the Nest–Tsygan theorem for the symplectic case, which are the results of Theorem 8.2 and Theorem 8.3, respectively. Finally, we show that the construction of the trace ${{\,\mathrm{Tr}\,}}^\mathcal {V}$ coincides with a trace map presented by Grady–Li–Li for cotangent targets, which is the statement of Proposition 9.3.

## Cyclic Formality

### The Kontsevich formality

Let $(\mathcal {T}_{poly}^\bullet (\mathbb {R}^d),[,]_{SN},{\mathrm {d}}=0)$ be the DGLA of multivector fields on $\mathbb {R}^d$ endowed with the Schouten–Nijenhuis bracket and the zero differential and let $(\mathcal {D}_{poly}^\bullet (\mathbb {R}^d),[,]_G,b)$ be the DGLA of multidifferential operators on $\mathbb {R}^d$ endowed with the Gerstenhaber bracket and the Hochschild differential. In [36] Kontsevich proved the celebrated formality theorem, which states that these two complexes are quasi-isomorphic as $L_\infty$-algebras.

#### Theorem 2.1

(Kontsevich [36]). There exists an $L_\infty$-quasi-isomorphism

1 $$\begin{aligned} \mathcal {U}:(\mathcal {T}_{poly}^\bullet (\mathbb {R}^d),[,]_{SN},{\mathrm {d}}=0)\longrightarrow (\mathcal {D}_{poly}^\bullet (\mathbb {R}^d),[,]_G,b). \end{aligned}$$

For the case of degree two, Theorem 2.1 implies a star product on $\mathbb {R}^d$ endowed with any Poisson structure. Moreover, Theorem 2.1 can be extended to a global version, where $\mathbb {R}^d$ can be replaced by any finite-dimensional manifold *M* as we will also describe in Sect. 4.6. Let us briefly recall the main objects to understand the ***formality theorem***.

#### The Hochschild complex and the Gerstenhaber bracket

Let *A* be a unital algebra with unit 1. One can consider the graded algebra $C_\bullet (A):=A\otimes {\bar{A}}^{\otimes \bullet }$, where ${\bar{A}}:=A/\mathbb {R}1$. This space is endowed with a map

2 $$\begin{aligned} b([a_0\otimes \cdots \otimes a_m])=\sum _{i=1}^{m-1}(-1)^{i}[a_0\otimes \cdots \otimes a_ia_{i+1}\otimes \cdots \otimes a_m]+(-1)^m[a_ma_0\otimes \cdots \otimes a_{m-1}].\nonumber \\ \end{aligned}$$

One can check that $b:C_\bullet (A)\longrightarrow C_{\bullet -1}(A)$ is a differential, called the ***Hochschild differential*** and the tuple $(C_\bullet (A),b)$ is called the ***Hochschild chain complex*** of *A*. Here we denote by $[a_0\otimes \cdots \otimes a_m]$ the class of $a_0\otimes \cdots \otimes a_m$ in $C_\bullet (A)$. Moreover, we define $C_m(A)=0$ for all $m<0$. The DGLA of multidifferential operators $\mathcal {D}_{poly}^\bullet (M)$, for a manifold *M*, can thus be seen as the subcomplex of the shifted complex $C^\bullet (A):={{\,\mathrm{Hom}\,}}(A^{\otimes \bullet +1},A)$, where $A=C^\infty (M)$, consisting of multilinear maps which are differential operators in each argument. The ***Gerstenhaber bracket*** of two multidifferential operators $D,D'$ is given by

3 $$\begin{aligned}{}[D,D']_G:=D\bullet _GD'-(-1)^{\vert D\vert \cdot \vert D'\vert }D'\bullet _GD, \end{aligned}$$

where $\vert D\vert$ denotes the degree of the multidifferential operator *D* and the ***Gerstenhaber product*** $\bullet _G$ is given by

4 $$\begin{aligned} D\bullet _G D':=\sum _{k=0}^n(-1)^{\vert D'\vert \cdot (\vert D\vert -k)}D\circ (\mathrm {id}^{\otimes k}\otimes D'\otimes \mathrm {id}^{\otimes \vert D\vert -k}). \end{aligned}$$

The differential on $C^\bullet (A)$ is given in terms of the Gerstenhaber bracket by $[\mu ,]_G$ for $\mu \in {{\,\mathrm{Hom}\,}}(A\otimes A,A)$ being the multiplication map of *A*. In fact, in [30] it was shown that the Hochschild cohomology $HH^\bullet (A)$ together with $\bullet _G$ and $[,]_G$ is a Gerstenhaber algebra.

#### Multivector fields and the Schouten–Nijenhuis bracket

The space of multivector fields on a manifold *M* is given by $\Gamma (\bigwedge ^\bullet TM)$. We define $\mathcal {T}^{\bullet }_{poly}(M):=\bigoplus _{j\ge -1}\Gamma (\bigwedge ^{j+1}TM)$, with the convention that $\mathcal {T}^{-1}_{poly}(M)=C^\infty (M)$, $\mathcal {T}^{0}_{poly}(M)=\Gamma (TM)$, $\mathcal {T}^{1}_{poly}(M)=\Gamma (\bigwedge ^2TM)$, etc. The ***Schouten–Nijenhuis bracket*** $[,]_{SN}$ is given by the usual Lie bracket extended to multivector fields by the Leibniz rule, i.e. for multivector fields $\alpha ,\beta ,\gamma$ we have

5 $$\begin{aligned} {[}\alpha \wedge \beta ,\gamma ]_{SN}=\alpha \wedge [\beta ,\gamma ]_{SN}+(-1)^{\vert \gamma \vert \cdot (\vert \beta \vert +1)}[\alpha ,\gamma ]_{SN}\wedge \beta . \end{aligned}$$

#### The Hochschild–Kostant–Rosenberg map

Consider vector fields $\xi _1,\ldots ,\xi _n\in \mathcal {T}_{poly}^0(M)$ and $f_1,\ldots ,f_n\in A$. One can construct a map, which for $n\ge 1$ is given by

6 $$\begin{aligned} \begin{aligned} \mathcal {T}_{poly}^{n-1}(M)&\longrightarrow \mathcal {D}_{poly}^{n-1}(M)\\ \xi _1\wedge \dots \wedge \xi _n&\longmapsto \left( f_1\otimes \cdots \otimes f_n\longmapsto \frac{1}{n!}\sum _{\sigma \in S_n}\text {sign}(\sigma )\xi _{\sigma (1)}(f_1)\cdots \xi _{\sigma (n)}(f_n)\right) , \end{aligned} \end{aligned}$$

and for $n=0$ it is given by the identity on $C^\infty (M)$. Here $S_n$ denotes the symmetric group of order *n*. This map is called ***Hochschild–Kostant–Rosenberg (HKR) map***. One can check that it is indeed a chain map and a quasi-isomorphism of complexes, but does not respect the Lie bracket on the level of complexes. In fact Kontsevich’s $L_\infty$-quasi-isomorphism $\mathcal {U}$ gives a solution to this problem as a certain extension of the HKR map. In particular, the first Taylor component $\mathcal {U}_1$ of $\mathcal {U}$ is precisely the HKR map.

### The Kontsevich–Tsygan formality

One can generalize the formality construction to a cyclic version by considering ***cyclic chains***. There is another differential, called the ***Connes differential*** [22, 23], of degree $+1$ on the Hochschild complex given by

7 $$\begin{aligned} B([a_0\otimes \cdots \otimes a_m]):=\sum _{i=0}^{m}(-1)^{im}[1\otimes a_i\otimes \cdots \otimes a_m\otimes a_0\otimes \cdots \otimes a_{i-1}]. \end{aligned}$$

Note that there is an HKR chain map

8 $$\begin{aligned} \begin{aligned} (C_\bullet (A),b)&\longrightarrow (\Omega ^\bullet (M,\mathbb {R}),{\mathrm {d}}=0)\\ [a_0\otimes \cdots \otimes a_m]&\longmapsto \frac{1}{m!}a_0{\mathrm {d}}a_1\wedge \cdots \wedge {\mathrm {d}}a_m. \end{aligned} \end{aligned}$$

This map is also called the ***Connes map*** [23], which identifies cyclic and de Rham cohomology. Following Getzler [32], the ***negative cyclic chain complex*** is then given by

9 $$\begin{aligned} CC_{-\bullet }^-(A):=C_{-\bullet }(A)[u] \end{aligned}$$

endowed with the differential $b+uB$. Here *u* denotes some formal variable of degree 2. Similarly to the negative cyclic chain complex, one can define the ***periodic cyclic chain complex*** by allowing negative powers of the formal parameter *u*, hence we have the formal Laurent polynomials $PC_{-\bullet }(A):=C_{-\bullet }(A)[u,u^{-1}]$. We can extend the HKR map by $\mathbb {R}[u]$-linearity and obtain a quasi-isomorphism

10 $$\begin{aligned} (CC^-_{-\bullet }(A),b+uB)\longrightarrow (\Omega ^{-\bullet }(M,\mathbb {R})[u],u{\mathrm {d}}). \end{aligned}$$

Consider a module *W* over the graded algebra $\mathbb {R}[u]$ of finite projective dimension and define $CC_{-\bullet }^W(A):=C_{-\bullet }(A)[u]\otimes _{\mathbb {R}[u]}W$. The formality for cyclic chains is given by the following theorem.

#### Theorem 2.2

[Kontsevich–Tsygan [42]]. There exists an $L_\infty$-quasi-isomorphism

11 $$\begin{aligned} \mathcal {U}^{cyc}:(CC_{-\bullet }^W(A),b+uB)\longrightarrow (\Omega ^{-\bullet }(M,\mathbb {R})[u]\otimes _{\mathbb {R}[u]}W,u{\mathrm {d}}). \end{aligned}$$

This was proven by Shoikhet, Willwacher and globally extended by Dolgushev using Fedosov resolution. Using Shoikhet’s $L_\infty$-quasi-isomorphism $\mathcal {U}^{Sh}$, one can obtain Theorem 2.2 as a corollary by obtaining $\mathcal {U}^{Sh}\circ b={\mathrm {d}}\circ \mathcal {U}^{Sh}$ [46].

#### Remark 2.3

This construction leads to a field theoretic construction using the Poisson Sigma Model on the disk as we will see in Sect. 6. One can construct a trace map which uses an $\mathbb {R}[u]$-linear morphism of $L_\infty$-modules over some suitable algebra.

## Fedosov’s Approach to Deformation Quantization

In this section we want to recall the most important notions and constructions of [26].

### Weyl algebra and Moyal product

Let $(M,\omega )$ be a symplectic manifold and let $\{x_i\}$ be local coordinates on *M* and $\{y^i\}$ coordinates on the corresponding fiber of the tangent bundle, i.e. $(x^{i},y^{i})\in M\times T_{x^i}M$. Consider the ***Weyl bundle***  associated to *M*, where  denotes the completed symmetric algebra. The Weyl bundle can be regarded as a deformation of the bundle of formal functions on $T^*M$. We will write $\mathcal {W}$ instead of $\mathcal {W}(M)$ whenever it is clear. A section $a\in \Gamma (\mathcal {W})$ is locally given by^2^

12 $$\begin{aligned} a(x,y,\hbar )=\sum _{k,\ell }\hbar ^k a_{k,i_1,\ldots ,i_\ell }(x)y^{i_1}\cdots y^{i_\ell }, \end{aligned}$$

where $a_{k,i_1,\ldots ,i_\ell }\in C^\infty (M)$. In each fiber $\mathcal {W}_x$ for $x\in M$, one can construct an algebra structure by considering the associative product

13 $$\begin{aligned} \begin{aligned} \star :\mathcal {W}_x\times \mathcal {W}_x&\longrightarrow \mathcal {W}_x,\\ (a(x,\hbar ),b(x,\hbar ))&\longmapsto (a\star b)(x,\hbar ):=\exp \left( -\frac{\mathrm {i}\hbar }{2}\omega ^{ij}\frac{\partial }{\partial y^{i}}\frac{\partial }{\partial z^{j}}\right) a(y,\hbar )b(z,\hbar )\Big |_{z=y}\\&\qquad = \sum _{k=0}^\infty \left( -\frac{\mathrm {i}\hbar }{2}\right) ^k\frac{1}{k!}\omega ^{i_1j_1}\cdots \omega ^{i_kj_k}\frac{\partial ^k a}{\partial y^{i_1}\cdots \partial y^{i_k}}\frac{\partial ^k b}{\partial z^{j_1}\cdots \partial z^{j_k}}. \end{aligned} \end{aligned}$$

Here we denote by $(\omega ^{ij})$ the components of the inverse $\omega ^{-1}$ of the symplectic form. For any $x\in M$, the tuple $(\mathcal {W}_x,\star )$ is called the ***Weyl algebra*** and $\star$ is called the ***Moyal product***. One can check that

14 $$\begin{aligned} \lim _{\hbar \rightarrow 0}\frac{1}{\hbar }(a\star b-b\star a)=\{a,b\}, \end{aligned}$$

where $\{,\}$ is the Poisson bracket coming from the symplectic structure $\omega$, which makes sure that $\star$ is actually a deformation quantization of $T^*_xM$ with constant Poisson structure $\omega _x^{-1}$. Let $\Omega ^\bullet (M,\mathcal {W})$ denote the space of global differential forms on *M* with values in $\mathcal {W}$. A section $a\in \Gamma (\Omega ^\bullet (M,\mathcal {W}))$ is of the form

15 $$\begin{aligned} a(x,y,{\mathrm {d}}x,\hbar )=\sum _{k,p,q}\hbar ^k a_{k,i_1,\ldots ,i_p,j_1,\ldots ,j_q}(x)y^{i_1}\cdots y^{i_p}{\mathrm {d}}x^{j_1}\wedge \cdots \wedge {\mathrm {d}}x^{j_q}, \end{aligned}$$

Moreover, we define the operators $\delta$ and $\delta ^*$ according to [26] by

16 $$\begin{aligned} \delta a:={\mathrm {d}}x^k\wedge \frac{\partial a}{\partial y^k},\qquad \delta ^* a:=y^k\iota _{\frac{\partial }{\partial x^k}}a. \end{aligned}$$

where $\iota$ denotes the contraction. Define $\delta ^{-1}:=\frac{1}{p+q}\delta ^*$ for $p+q>0$ and zero if $p+q=0$.

### Symplectic connection and curvature

Consider now a symplectic connection $\nabla ^{TM}$ on the tangent bundle *TM*, i.e. a torsion-free connection such that $\nabla ^{TM}\omega =0$. This induces directly a connection $\nabla ^\mathcal {W}$ on $\mathcal {W}$ which we will just denote by $\nabla$. The curvature of this connection is given by

17 $$\begin{aligned} F^\nabla =\frac{1}{2}F^{i}_{jk\ell }{\mathrm {d}}x^{k}\wedge {\mathrm {d}}x^\ell . \end{aligned}$$

Moreover, consider the tensor

18 $$\begin{aligned} F:=\frac{1}{4}F_{ijk\ell }y^{i}y^j{\mathrm {d}}x^k\wedge {\mathrm {d}}x^\ell ,\quad F_{ijk\ell }:=\omega _{im} F_{jk\ell }^m. \end{aligned}$$

In [26] it was shown that the curvature of $\nabla$ can be formulated as

19 $$\begin{aligned} \nabla ^2=\frac{1}{\hbar }[F,]_\star , \end{aligned}$$

where $[,]_\star$ denotes the commutator with respect to the Moyal product $\star$.

### Fedosov’s main theorems

Consider a connection

20 $$\begin{aligned} \bar{\nabla } :=\nabla +\frac{1}{2\hbar }[\gamma ,]_\star \end{aligned}$$

on $\mathcal {W}$, where $\gamma \in \Omega ^1(M,\mathcal {W})$. One can check that $\bar{\nabla }$ is compatible with the Moyal product, i.e.

21 $$\begin{aligned} \bar{\nabla }(a\star b)=\bar{\nabla }(a)\star b+ a\star \bar{\nabla }(b). \end{aligned}$$

#### Theorem 3.1

[Fedosov [26]]. Consider a sequence $\{\omega _k\}_{k\ge 1}$ of closed 2-forms on *M*. Then there is a flat connection $\bar{\nabla }$ (that is $\bar{\nabla }^2=0$) defined as in (20) such that $\gamma =\sum _{i,j}\omega _{ij}y^{i}{\mathrm {d}}x^j+r$, where $r\in \Omega ^1(M,\mathcal {W})$ satisfying $\delta ^{-1}r=0$. Moreover, $\gamma$ satisfies

22 $$\begin{aligned} \bar{\nabla }\gamma =\nabla \gamma +\frac{1}{2\hbar }[\gamma ,\gamma ]_\star +F=\omega _\hbar ,\quad \omega _\hbar :=-\omega +\sum _{k\ge 1}\hbar ^k\omega _k. \end{aligned}$$

Consider the ***symbol map***

23 $$\begin{aligned} \begin{aligned} \sigma :\Gamma (\mathcal {W})&\longrightarrow C^\infty (M)[\![ \hbar ]\!]\\ a(x,y,\hbar )=\sum _{k,\ell }\hbar ^k a_{k,i_1,\ldots ,i_\ell }(x)y^{i_1}\cdots y^{i_\ell }&\longmapsto a(x,0,\hbar )=\sum _{k}\hbar ^k a_{k,i_1,\ldots ,i_\ell }(x), \end{aligned} \end{aligned}$$

which sends all the $y^{i}$s to zero.

#### Theorem 3.2

[Fedosov [26]]. The symbol map induces an isomorphism

24 $$\begin{aligned} \sigma :H^0_\nabla (\Gamma (\mathcal {W}))\xrightarrow {\sim }C^\infty (M)[\![ \hbar ]\!], \end{aligned}$$

where $H^0_\nabla (\Gamma (\mathcal {W}))$ denotes the space of flat sections of the Weyl bundle with respect to $\nabla$. Moreover, since for any flat connection Equation (21) holds, we can construct a global star product on $C^\infty (M)[\![ \hbar ]\!]$ by the formula

25 $$\begin{aligned} f\star _Mg:=\sigma (\sigma ^{-1}(f)\star \sigma ^{-1}(g)), \end{aligned}$$

which defines a deformation quantization on $(M,\omega )$.

#### Remark 3.3

Theorem 3.2 tells us the existence of a global version of the Moyal product for symplectic manifolds. There is a similar approach to globalization for any Poisson manifold, where we start with Kontsevich’s star product on the local picture using elements of formal geometry, such as the construction of the Grothendieck connection. A modification (deformed version) of this connection will replace the symplectic connection in Fedosov’s picture. In fact, Fedosov’s construction uses the exponential map of a symplectic connection, whereas the more general approach uses the notion of a formal exponential map as we will discuss in the next section.

## Formal Geometry and Grothendieck Connection

In this section we want to recall the most important notions of formal geometry as in [9, 29], the construction of the Grothendieck connection, its deformed version and the relation to Fedosov’s quantization approach for the case of a symplectic manifold [8, 15, 17–19, 21].

### Formal exponential maps

Let *M* be a smooth manifold. Let $\varphi :U \longrightarrow M$ where $U \subset TM$ is an open neighbourhood of the zero section. For $x \in M, y \in T_xM \cap U$ we write $\varphi _x(y):=\varphi (x,y)$. We say that $\varphi$ is a ***generalized exponential map*** if for all $x \in M$ we have that $\varphi _x(0) = x$, and ${\mathrm {d}}\varphi _x\vert _{y=0} = \mathrm {id}_{T_xM}$. In local coordinates we can write

26 $$\begin{aligned} \varphi _x^{i}(y)=x^{i}+y^{i}+\frac{1}{2}\varphi _{x,jk}^{i}y^jy^k+\frac{1}{3!}\varphi ^{i}_{x,jk\ell }y^jy^ky^\ell +\cdots \end{aligned}$$

where the $x^i$ are coordinates on the base and the $y^i$ are coordinates on the fibers. We identify two generalized exponential maps if their jets at $y=0$ agree to all orders. A ***formal exponential map*** is an equivalence class of generalized exponential maps. It is completely specified by the sequence of functions $\left( \varphi ^i_{x,i_1,\ldots ,i_k}\right) _{k=0}^{\infty }$. By abuse of notation, we will denote equivalence classes and their representatives by $\varphi$. From a formal exponential map $\varphi$ and a function $f \in C^{\infty }(M)$, we can produce a section  by defining $\sigma _x = \textsf {T}\varphi _x^*f$, where $\textsf {T}$ denotes the Taylor expansion in the fiber coordinates around $y=0$ and we use any representative of $\varphi$ to define the pullback. We denote this section by $\textsf {T}\varphi ^*f$; it is independent of the choice of representative, since it only depends on the jets of the representative.

#### Example 4.1

The exponential map of a connection is an example of an exponential map.

### The Grothendieck connection

As it was shown [8, 9, 15, 17, 29], one can define a flat connection *D* on  with the property that $D\sigma = 0$ if and only if $\sigma = \textsf {T}\varphi ^*f$ for some $f\in C^\infty (M)$. Namely, $D = {\mathrm {d}}_x + L_R$ where  is a 1-form with values in derivations of , which we identify with . We have denoted by ${\mathrm {d}}_x$ the de Rham differential on *M* and by *L* the Lie derivative. In coordinates we have

27 $$\begin{aligned} R(\sigma )_\ell =-\frac{\partial \sigma }{\partial y^j}\left( \left( \frac{\partial \varphi }{\partial y}\right) ^{-1}\right) _k^j\frac{\partial \varphi ^k}{\partial x^\ell }. \end{aligned}$$

Define $$R(x,y):=R_\ell (x,y){\mathrm {d}}x^\ell $$, $$R_\ell (x,y):=R_\ell ^j(x,y)\frac{\partial }{\partial y^j}$$, $$R^j(x,y):=R^j_\ell (x,y){\mathrm {d}}x^\ell $$, and

28 $$\begin{aligned} R_\ell ^j=-\left( \left( \frac{\partial \varphi }{\partial y}\right) ^{-1}\right) _k^j\frac{\partial \varphi ^k}{\partial x^\ell }=-\delta _\ell ^j+O(y). \end{aligned}$$

For , $L_R\sigma$ is given by the Taylor expansion (in the *y* coordinates) ofwhere we denote by ${\mathrm {d}}_y$ the de Rham differential on the fiber. This shows that *R* does not depend on the choice of coordinates. One can generalize this also for any fixed vector $\xi = \xi ^{i}(x) \frac{\partial }{\partial x^i}\in T_xM$ by29where30Here $\xi (x)$ would replace the 1-form part ${\mathrm {d}}x^{i}$. The connection *D* is called the ***Grothendieck connection***. Note that its flatness is equivalent to the Maurer–Cartan equation

31 $$\begin{aligned} {\mathrm {d}}_xR + \frac{1}{2}[R,R] = 0. \end{aligned}$$

Moreover, using the Poincaré lemma on $T_xM$ it can be shown that its cohomology is concentrated in degree 0 and is given by32

### Lifting formal exponential maps to cotangent bundles

We want to consider the case were our manifold is given by a cotangent bundle.

#### Proposition 4.2

If the base manifold is given by a cotangent bundle $T^*M$, the vector field ${\bar{R}}$, defined by the lift of the formal exponential map, is linear in the fiber coordinate of $T_{(q,p)}T^*M$ for any $(q,p)\in T^*M$.

#### Proof

Let *M* be a smooth manifold and consider a formal exponential map $\varphi :TM\longrightarrow M$. Moreover, let $\bar{\varphi }:TT^*M\longrightarrow T^*M$ be the lift of the formal exponential map to the cotangent bundle of *M*. Explicitly, for $(q,p)\in T^*M$, we have

$$\begin{aligned} \bar{\varphi }_{(q,p)}:T_{(q,p)}T^*M\cong T_qT_q^*M\oplus T_pT_q^*M\cong T_qM\oplus T_q^*M\longrightarrow T^*M. \end{aligned}$$

Let $({\bar{q}},{\bar{p}})\in T_{(q,p)}T^*M$, and hence ${\bar{q}}\in T_qM$ and ${\bar{p}}\in T_q^*M$. Note that $\varphi _q:T_qM\longrightarrow M$, and thus

$$\begin{aligned} \left( {\mathrm {d}}_{{\bar{q}}}(\varphi _{q})\right) ^{*,-1}:T_q^*M\longrightarrow T^*_{\varphi _q({\bar{q}})}M, \end{aligned}$$

since ${\mathrm {d}}_{{\bar{q}}}(\varphi _q):T_{{\bar{q}}}T_qM\cong T_qM\longrightarrow T_{\varphi _q({\bar{q}})}M$. Then we can write the lift of the exponential map as

$$\begin{aligned} \bar{\varphi }_{(q,p)}({\bar{q}},{\bar{p}})=\left( \varphi _q({\bar{q}}),\left( {\mathrm {d}}_{{\bar{q}}}(\varphi _{q})\right) ^{*,-1}{\bar{p}}\right) \in T^*M. \end{aligned}$$

For $x=(q,p)\in T^*M$ and $y=({\bar{q}},{\bar{p}})\in T_xT^*M=T_{(q,p)}T^*M$, we want to compute

$$\begin{aligned} \left( {\mathrm {d}}_y(\bar{\varphi }_x)\right) ^{-1},\qquad {\mathrm {d}}_x(\bar{\varphi }_x). \end{aligned}$$

We write $\bar{\varphi }^{{\bar{q}}}:=\varphi _{q}({\bar{q}})$ and $\bar{\varphi }^{{\bar{p}}}:=\left( {\mathrm {d}}_{\bar{q}}(\varphi _{q})\right) ^{*,-1}{\bar{p}}$. Hence we get

33 $$\begin{aligned} {\mathrm {d}}_y\bar{\varphi }_x=\begin{pmatrix}\frac{\partial \bar{\varphi }^{\bar{q}}}{\partial {\bar{q}}}&{}\frac{\partial \bar{\varphi }^{{\bar{q}}}}{\partial {\bar{p}}}\\ \frac{\partial \bar{\varphi }^{{\bar{p}}}}{\partial {\bar{q}}}&{} \frac{\partial \bar{\varphi }^{{\bar{p}}}}{\partial \bar{p}}\end{pmatrix}=\begin{pmatrix} \frac{\partial \bar{\varphi }^{{\bar{q}}}}{\partial {\bar{q}}}&{} \varvec{0}\\ \frac{\partial \bar{\varphi }^{{\bar{p}}}}{\partial {\bar{q}}}&{} \frac{\partial \bar{\varphi }^{{\bar{p}}}}{\partial {\bar{p}}} \end{pmatrix},\qquad {\mathrm {d}}_x\bar{\varphi }_x=\begin{pmatrix}\frac{\partial \bar{\varphi }^{\bar{q}}}{\partial q}&{} \frac{\partial \bar{\varphi }^{{\bar{p}}}}{\partial p}\\ \frac{\partial \bar{\varphi }^{{\bar{p}}}}{\partial q}&{}\frac{\partial \bar{\varphi }^{{\bar{p}}}}{\partial p}\end{pmatrix}=\begin{pmatrix}\frac{\partial \bar{\varphi }^{\bar{q}}}{\partial q}&{}\varvec{0}\\ \frac{\partial \bar{\varphi }^{\bar{p}}}{\partial q}&{}\varvec{0}\end{pmatrix}. \end{aligned}$$

Moreover, we have

34 $$\begin{aligned} \left( {\mathrm {d}}_y\bar{\varphi }_x\right) ^{-1}=\begin{pmatrix}\left( \frac{\partial \bar{\varphi }^{{\bar{q}}}}{\partial {\bar{q}}}\right) ^{-1}&{} \varvec{0}\\ -\left( \frac{\partial \bar{\varphi }^{{\bar{p}}}}{\partial {\bar{p}}}\right) ^{-1}\circ \left( \frac{\partial \bar{\varphi }^{{\bar{p}}}}{\partial {\bar{q}}}\right) \circ \left( \frac{\partial \bar{\varphi }^{{\bar{q}}}}{\partial {\bar{q}}}\right) ^{-1}&{}\left( \frac{\partial \bar{\varphi }^{{\bar{p}}}}{\partial {\bar{p}}}\right) ^{-1}\end{pmatrix}. \end{aligned}$$

Thus, we get

35 $$\begin{aligned} -\left( {\mathrm {d}}_y\bar{\varphi }_x\right) ^{-1}\circ {\mathrm {d}}_x\bar{\varphi }_x=\begin{pmatrix}-\left( \frac{\partial \bar{\varphi }^{\bar{q}}}{\partial {\bar{q}}}\right) ^{-1}\circ \left( \frac{\partial \bar{\varphi }^{\bar{q}}}{\partial q}\right) &{}\varvec{0}\\ \left( \frac{\partial \bar{\varphi }^{\bar{p}}}{\partial {\bar{p}}}\right) ^{-1}\circ \left( \frac{\partial \bar{\varphi }^{\bar{p}}}{\partial {\bar{q}}}\right) \circ \left( \frac{\partial \bar{\varphi }^{\bar{q}}}{\partial {\bar{q}}}\right) ^{-1}\circ \left( \frac{\partial \bar{\varphi }^{\bar{q}}}{\partial q}\right) -\left( \frac{\partial \bar{\varphi }^{{\bar{p}}}}{\partial \bar{p}}\right) ^{-1}\circ \left( \frac{\partial \bar{\varphi }^{{\bar{p}}}}{\partial q}\right) &{}\varvec{0}\end{pmatrix}. \end{aligned}$$

If we consider the lift ${\bar{R}}=-{\mathrm {d}}_y\circ \left( {\mathrm {d}}_y\bar{\varphi }_x\right) ^{-1}\circ {\mathrm {d}}_x\bar{\varphi }_x$, we get that ${\bar{R}}$ is linear in ${\bar{p}}$ as claimed. $\quad \square$

#### Remark 4.3

Proposition 4.2 will simplify the graphs in the Feynman graph expansion of the formal global Poisson Sigma Model for cotangent targets as we will see later on.

### The deformed Grothendieck connection

Let $M\subset \mathbb {R}^d$ be an open subset and consider a Poisson structure $\pi$ on $\mathbb {R}^d$. We will denote its associated Weyl bundle^3^ by  similarly as in the symplectic case. Using Kontsevich’s formality map, one can construct a global connection $\mathcal {D}$ on $\Gamma (\mathcal {W})$ as follows: For some vector field $\xi$, define a differential operator

36 $$\begin{aligned} A(\xi ,\pi ):=\sum _{j=1}^\infty \frac{\hbar ^j}{j!}\mathcal {U}_{j+1}(\xi ,\pi ,\ldots ,\pi )\in \mathcal {D}_{poly}^0(M), \end{aligned}$$

using the formality map $\mathcal {U}$. Define the quantized version $\mathcal {D}$ of *D* by replacing  by  in (29), where we consider a fixed vector $\xi \in T_xM$. Hence we have37This connection can be extended to a well-defined global connection $\mathcal {D}$ on $\mathcal {W}$. It is in fact given as a deformation of *D*, i.e. $\mathcal {D}=D+O(\hbar )$. Moreover, $\mathcal {D}$ is not flat but one can check that it is an inner derivation as in (19). Let $\star$ denote Kontsevich’s star product. For any section $\sigma \in \Gamma (\mathcal {W})$ we have

38 $$\begin{aligned} \mathcal {D}^2\sigma =[F,\sigma ]_\star :=F\star \sigma -\sigma \star F, \end{aligned}$$

where $F\in \Omega ^2(M,\mathcal {W})$ denotes the Weyl curvature tensor of $\mathcal {D}$, which can be also expressed by Kontsevich’s $L_\infty$-morphism. For two vector fields $\xi ,\zeta$, define a function

39 $$\begin{aligned} F_0(\xi ,\zeta ,\pi ):=\sum _{j=1}^\infty \frac{\hbar ^j}{j!}\mathcal {U}_{j+2}(\xi ,\zeta ,\pi ,\ldots ,\pi )\in \mathcal {D}_{poly}^0(M)\cong C^\infty (M), \end{aligned}$$

in terms of the $L_\infty$-morphism $\mathcal {U}$. Then we can define the Weyl curvature tensor of $\mathcal {D}$ to be given by

40 $$\begin{aligned} F(\xi ,\zeta ):=F_0(\xi ,\zeta ,\mathsf {T}\varphi _x^*\pi ). \end{aligned}$$

Moreover, one can check that the Bianchi identity $\mathcal {D}F=0$ holds and that for any $\gamma \in \Omega ^1(M,\mathcal {W})$ the map

41 $$\begin{aligned} \bar{\mathcal {D}}:= \mathcal {D}+[\gamma ,]_\star \end{aligned}$$

is a derivation, i.e. $\bar{\mathcal {D}}(\sigma \star \tau )=\bar{\mathcal {D}}(\sigma )\star \tau +\sigma \star \bar{\mathcal {D}}(\tau )$ for all $\sigma ,\tau \in \Gamma (\mathcal {W})$. Computing $\bar{\mathcal {D}}^2$ directly, one can see that the Weyl curvature tensor ${\bar{F}}$ of $\bar{\mathcal {D}}$ is given by

42 $$\begin{aligned} {\bar{F}}=F+\mathcal {D}\gamma +\gamma \star \gamma . \end{aligned}$$

#### Proposition 4.4

There exists a $\gamma \in \Omega ^1(M,\mathcal {W})$ such that ${\bar{F}}=0$. More generally, for any $\omega _\hbar =\omega _0+\hbar \omega _1+\hbar ^2\omega _2+\cdots \in \Omega ^2(M,\mathcal {W})$ with $\mathcal {D}\omega _\hbar =0$ and $[\omega _\hbar ,]_\star =0$, there exists a $\gamma \in \Omega ^1(M,\mathcal {W})$ such that

43 $$\begin{aligned} \bar{F}=F+\mathcal {D}\gamma +\gamma \star \gamma =\omega _\hbar . \end{aligned}$$

It is clear that (42) is the special case of (43) for $\omega _\hbar =0$. Proposition 4.4 can be shown by using techniques of homological perturbation theory. Note that the Bianchi identity for ${\bar{F}}$ implies that $\bar{\mathcal {D}}\omega _\hbar =\mathcal {D}\omega _\hbar =0$ if $\omega _\hbar$ is a central element of the Weyl algebra endowed with Kontsevich’s star product. Equation (43) can be seen as a more general version of (22) for Poisson manifolds, where $\mathcal {D}$ takes the place of the symplectic connection $\nabla$ and $\bar{\mathcal {D}}$ the one of $\bar{\nabla }$. We will say that a connection is ***compatible*** if its extension to differential forms $\Omega ^\bullet (M,\mathcal {W})$ is a derivation of degree $+1$ with respect to the star product on the Weyl algebra. A compatible connection on $\Gamma (\mathcal {W})$ is called a ***Fedosov connection*** if it is an inner derivation with respect to its Weyl curvature tensor and it satisfies the Bianchi identity. By the constructions above, the deformed Grothendieck connection $\mathcal {D}$ is a Fedosov connection as well as any symplectic connection $\nabla$ on the tangent bundle of a symplectic manifold as in Fedosov’s construction. Note that, as we have seen, if $\mathcal {D}$ is a Fedosov connection, then $\bar{\mathcal {D}}=\mathcal {D}+[\gamma ,]_\star$ is also a Fedosov connection.

### Grothendieck connection on symplectic manifolds

Let $(M,\omega )$ be a symplectic manifold which can be considered as a special case of a Poisson manifold with Poisson structure $\pi$ coming from the symplectic form. By Darboux’s theorem, we consider a constant symplectic form $\omega ^\varphi :=\varphi ^*_x\omega$ lifted to the formal construction for any $x\in M$. Note that in this case *R* is a 1-form on *M* with values in formal Hamiltonian vector fields^4^ for the corresponding Hamiltonian functions $h_x$ such that $h_x|_{y=0}=0$. For any $x\in M$, $h_x$ is a 1-form with values in  and for any section $\sigma \in \Gamma (\mathcal {W})$ we get $\mathcal {D}\sigma _x={\mathrm {d}}_x\sigma _x+\frac{1}{2\hbar }[h_x,\sigma _x]_\star$. For the Weyl curvature tensor we get $F_x(\xi ,\zeta )=\frac{1}{4\hbar ^2}([\langle h_x,\xi \rangle ,\langle h_x,\zeta \rangle ]_\star -2\hbar \{\langle h_x,\zeta \rangle ,\langle h_x,\zeta \rangle \})$. Consider a symplectic connection $\nabla$ on *TM*, which induces a connection $\mathcal {D}$ on $\Gamma (\mathcal {W})$, which acts as a derivation on the Weyl algebra. Its curvature is then given by $\mathcal {D}^2=\frac{1}{2\hbar }[F,]$ with $F\in \Omega ^2(M,\mathcal {W})$ given by $F=-\frac{1}{2}\omega (\nabla ^2y,y)$ is the quadratic form on *TM* associated to the curvature $\nabla ^2$ of $\nabla$. Fedosov showed that for a closed 2-form $\omega _\hbar =-\frac{1}{2\hbar }\omega +\omega _0+\hbar \omega _1+\hbar ^2\omega _2+\cdots \in \Omega ^2(M,\mathbb {R})[\![ \hbar ]\!]$ the equation

44 $$\begin{aligned} \nabla ^2+\nabla \gamma _\hbar +\gamma _\hbar \star \gamma _\hbar =\omega _\hbar \end{aligned}$$

has a solution $\gamma _\hbar =-\frac{1}{2\hbar }\omega _{ij}y^{i}{\mathrm {d}}x^j+\gamma _0+\hbar \gamma _1+\hbar ^2\gamma _2+\cdots \in \Omega ^1(M,\mathcal {W})$ such that $\gamma _\hbar |_{y=0}=0$. Moreover, we consider the formal exponential map coming from the symplectic connection $\nabla$

45 $$\begin{aligned} \varphi _x^{i}(y)=x^i+y^{i}+\frac{1}{2}\sum _{k,\ell }\Gamma _{k\ell }^{i}y^ky^\ell +\cdots \end{aligned}$$

Then the connection $\nabla +[\gamma _\hbar ,]_\star$ is given by $\bar{\mathcal {D}}=\mathcal {D}+[\gamma ,]_\star$ with $\gamma _\hbar =-\frac{1}{2\hbar }h_x+\gamma$, where $\gamma$ is a solution of (43) with $\omega _\hbar =\omega _0+\hbar \omega _1+\hbar ^2\omega _2+\cdots$. The star product constructed in this way, using a closed two form $\omega _\hbar \in \Omega ^2(M,\mathbb {R})[\![ \hbar ]\!]$, is equivalent to the one constructed by Fedosov associated to the class $-\frac{1}{2\hbar }\omega +\omega _\hbar$. Note that the deformations of the symplectic form are in one-to-one correspondence with their characteristic classes, which are formal power series $\omega _\hbar =\omega _0+\hbar \omega _1+\hbar ^2\omega _2+\cdots$, with $\omega _i\in H^2(M,\mathbb {R})$ such that $-\omega _0$ is the class of the symplectic form $\omega$. For more details on these constructions see [18].

### Globalization of Kontsevich’s star product

Consider again a Poisson manifold $(M,\pi )$. As already mentioned, the algebra of smooth functions on *M* is isomorphic to the subalgebra of *D*-closed sections of . Denote by $H^0_{\bar{\mathcal {D}}}(\Gamma (\mathcal {W}))$ the subalgebra of $\Gamma (\mathcal {W})$ consisting of $\bar{\mathcal {D}}$-closed sections of $\mathcal {W}$. Since *D* and $\bar{\mathcal {D}}$ are flat connections we have natural cochain complexes  and $(\Gamma (\mathcal {W}),\bar{\mathcal {D}})$.

#### Proposition 4.5

The subalgebra $H^0_{\bar{\mathcal {D}}}(\Gamma (\mathcal {W}))$ provides a deformation quantization of $(M,\pi )$.

More precisely, we can construct a ***cochain map***46which implies a ***quantization map***47This map induces an isomorphism $C^\infty (M)[\![ \hbar ]\!]\xrightarrow {\sim }H^0_{\bar{\mathcal {D}}}(\Gamma (\mathcal {W}))$, since there are no cohomological obstructions. Note that this is the analogue of the symbol map as in Fedosov’s quantization. Moreover, there is a unique $\rho$ for each $\bar{\mathcal {D}}$ such that $\rho |_{y=0}=\mathrm {id}$. Using this map, one can define a global version of Kontsevich’s star product, defined on the whole Poisson manifold *M* by

48 $$\begin{aligned} f \star _M g:=[\rho ^{-1}(\rho (\mathsf {T}\varphi ^* f)\star \rho (\mathsf {T}\varphi ^*g))]\Big |_{y=0}. \end{aligned}$$

Indeed, the map $\rho$ sends *D*-flat sections to $\bar{\mathcal {D}}$-flat sections since $\rho$ is a cochain map, i.e. we have $\rho \circ D=\bar{\mathcal {D}}\circ \rho$, and by compatibility with the star product, one can obtain that $J:=\rho (\mathsf {T}\varphi ^* f)\star \rho (\mathsf {T}\varphi ^*g)$ is again $\bar{\mathcal {D}}$-closed because $\mathsf {T}\varphi ^*f$ is *D*-closed for all $f\in C^\infty (M)[\![ \hbar ]\!]$. But since *J* is $\bar{\mathcal {D}}$-closed, we know that it has to lie in the image of $\rho$. Hence there exists some  such that $\rho (j)=J$. This implies that *j* is *D*-closed and thus of the form $j=\mathsf {T}\varphi ^*\tilde{j}$ for some ${\tilde{j}}\in C^\infty (M)[\![ \hbar ]\!]$. Setting the formal variables $y=0$ one finds a global construction for the star product.

This approach generalizes Fedosov’s construction for the Moyal product, to the globalization of Kontsevich’s star product. It can be translated into field theoretic concepts using the Grothendieck connection together with the Poisson Sigma Model as we will also briefly recall in Sect. 5.4.

## The Poisson Sigma Model and its Globalization

### The classical model

The data for the ***Poisson Sigma Model*** consists of a Poisson manifold $(M,\pi )$, a compact, connected 2-manifold $\Sigma$ (possibly with boundary), a map $X:\Sigma \longrightarrow M$, a 1-form $\eta \in \Gamma (\Sigma ,T^*\Sigma \otimes X^*T^*M)=\Omega ^1(\Sigma ,X^*T^*M)$, and an action functional

49 $$\begin{aligned} S_\Sigma (X,\eta )=\int _\Sigma \left( \langle \eta ,{\mathrm {d}}X\rangle +\frac{1}{2}\langle \pi (X),\eta \wedge \eta \rangle \right) . \end{aligned}$$

We consider the space of fields as vector bundle maps $F_\Sigma ={{\,\mathrm{Map}\,}}_{\text {VecBun}}(T\Sigma ,T^*M)$, i.e. we have the following diagram 
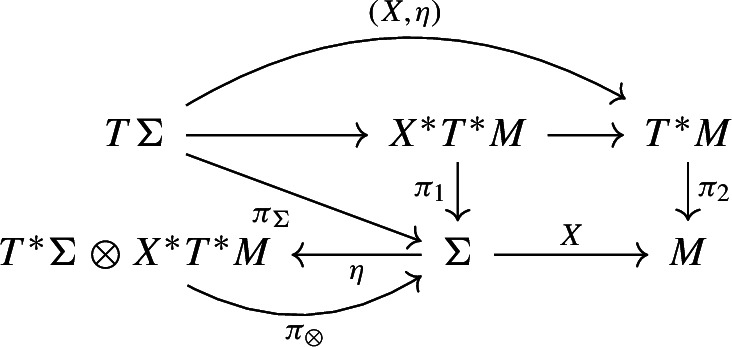
 Consider now the case where $\partial \Sigma \not =\varnothing$ and let $\iota _{\partial \Sigma }:\partial \Sigma \hookrightarrow \Sigma$ denote the inclusion of the boundary. Then we set the boundary conditions such that $\iota ^*_{\partial \Sigma }\eta =0$. This is convenient to choose, since the Euler–Lagrange equations are given by

50 $$\begin{aligned} {\mathrm {d}}X^{i}+\pi ^{ij}(X)\eta _j=0,\qquad {\mathrm {d}}\eta _i+\frac{1}{2}\partial _i\pi ^{jk}(X)\eta _j\wedge \eta _k=0. \end{aligned}$$

**Fig. 1:**
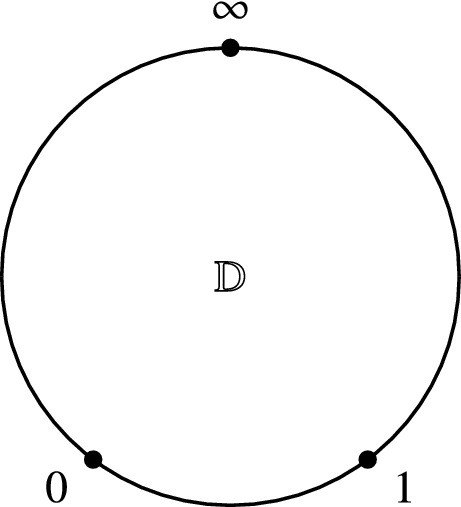
Cyclically ordered points on $S^1=\partial \mathbb {D}$

Hence, it is easy to consider the solution where $X=const.$ and $\eta =0$. In [11] it was shown that this model is directly connected to Kontsevich’s star product as formulating it by a quantum field theory where the space-time manifold $\Sigma$ is modelled by the disk $\mathbb {D}=\{x\in \mathbb {R}^2\mid \Vert x\Vert \le 1\}$. If we choose three points $0,1,\infty$ on the boundary $\partial \mathbb {D}$ counterclockwise (i.e. if we move from 0 counterclockwise on the boundary, we will first meet 1 and then $\infty$, see Fig. 1), Kontsevich’s star product is given by the semiclassical expansion of the ***path integral*** modelled by the Poisson Sigma Model as

51 $$\begin{aligned} f\star g(x)=\int _{X(\infty )=x}f(X(1))g(X(0))\exp \left( \frac{\mathrm {i}}{\hbar }S_{\mathbb {D}}(X,\eta )\right) . \end{aligned}$$

### BV formulation

The ***Batalin–Vilkovisky (BV) formalism*** [4–6] is a way of dealing with gauge theories^5^, i.e. of theories where the action is invariant under certain symmetries. There we usually associate to a space-time manifold $\Sigma$ a BV space of fields $\mathcal {F}_\Sigma$ (in general, if one starts with the BRST formalism, we get $\mathcal {F}_{\text {BV}}=T^*[-1]\mathcal {F}_{\text {BRST}}$), which is a $\mathbb {Z}$-graded ***supermanifold***, endowed with a $(-1)$-shifted symplectic structure $\omega _\Sigma$ and an action functional $S_\Sigma \in \mathcal {O}(\mathcal {F}_\Sigma )$ of degree 0 such that $\{S_\Sigma ,S_\Sigma \}=0$ (***Classical Master Equation***), where $\{,\}$ denotes the ***BV bracket*** coming from the odd symplectic form $\omega _\Sigma$. Here we denote by $\mathcal {O}(X)$ functions on a space *X*. We would like our theory to be local, i.e. we require the action to be given as an integral over some Lagrangian density $\mathscr {L}$ depending on fields and higher derivatives

52 $$\begin{aligned} S(\phi )=\int _\Sigma \mathscr {L}(\phi ,\partial \phi ,\ldots ), \quad \phi \in \mathcal {F}_\Sigma . \end{aligned}$$

Moreover, we consider the ***BV Laplacian*** $\Delta$ [35, 39], acting on functions on $\mathcal {F}_\Sigma$. We will denote by $\mathcal {O}_{\text {loc}}(\mathcal {F}_\Sigma )$ the space of such local functions on $\mathcal {F}_\Sigma$. One can check that $(\mathcal {O}_{\text {loc}}(\mathcal {F}_\Sigma ),\Delta )$ is a ***BV algebra*** (see Appendix A)^6^. Moreover, we can define a cohomological vector field (similarly as in the linear case, which would be the usual BRST charge) as the degree $+1$ Hamiltonian vector field $Q_\Sigma$ of $S_\Sigma$, i.e. $\iota _{Q_\Sigma }\omega _\Sigma =-{\mathrm {d}}_{\mathcal {F}_\Sigma }S_\Sigma$. Then we have $[Q_\Sigma ,Q_\Sigma ]=0$ and $Q_\Sigma =\{S_\Sigma ,\}$. Here ${\mathrm {d}}_{\mathcal {F}_\Sigma }$ denotes the de Rham differential on the BV space of fields $\mathcal {F}_\Sigma$.

#### BV formulation for the Poisson sigma model

Let everything be as in the setting of the Poisson Sigma Model. The BV space of fields is given by $\mathcal {F}_\Sigma ={{\,\mathrm{Map}\,}}_{\text {SupMnf}}(T[1]\Sigma ,T^*[1]M)$ which are maps between supermanifolds, where for the superfields $(\mathsf {X},\varvec{\eta })\in \mathcal {F}_\Sigma$ we have the ***BV action functional***

53 $$\begin{aligned} S_{\Sigma }(\mathsf {X},\varvec{\eta })=\int _{T[1]\Sigma }\left( \langle \varvec{\eta },\varvec{D}\mathsf {X}\rangle +\frac{1}{2}\langle \pi (\mathsf {X}),\varvec{\eta }\wedge \varvec{\eta }\rangle \right) , \end{aligned}$$

where $\varvec{D}=\theta ^\mu \frac{\partial }{\partial x^\mu }$ is the superdifferential for even coordinates $(x^\mu )$ and odd coordinates $(\theta ^\mu )$ and $\langle ,\rangle$ denotes the pairing of tangent and cotangent space of *M*. One can write out the components of the superfields in terms of fields, antifields and ghosts as follows

54 $$\begin{aligned} \mathsf {X}^{i}&=X^{i}+\eta ^{+,i}_\mu \theta ^{\mu }+\frac{1}{2}\beta ^{+,i}_{\mu \nu }\theta ^{\mu }\theta ^{\nu }, \end{aligned}$$

55 $$\begin{aligned} \varvec{\eta }_i&=\beta _{i}+\eta _{i,\mu }\theta ^{\mu }+\frac{1}{2}X^+_{i,\mu \nu }\theta ^{\mu }\theta ^{\nu }, \end{aligned}$$

where $\beta$ denotes the ghost field. For a field $\phi$ we denote by $\phi ^+$ its antifield. Note that we have the relation ${{\,\mathrm{gh}\,}}(\phi )+{{\,\mathrm{gh}\,}}(\phi ^+)=-1$ and $\deg (\phi )+\deg (\phi ^+)=2$, where “${{\,\mathrm{gh}\,}}$” denotes the ***ghost number*** which corresponds to the $\mathbb {Z}$-grading on $\mathcal {F}_\Sigma$, and “$\deg$” denotes the form degree. Thus we get

$$\begin{aligned} \deg (X)&=0,\quad \deg (X^+)=2,\quad {{\,\mathrm{gh}\,}}(X)=0,\quad {{\,\mathrm{gh}\,}}(X^+)=-1\\ \deg (\eta )&=1,\quad \deg (\eta ^+)=1,\quad {{\,\mathrm{gh}\,}}(\eta )=0,\quad {{\,\mathrm{gh}\,}}(\eta ^+)=-1\\ \deg (\beta )&=0,\quad \deg (\beta ^+)=2,\quad {{\,\mathrm{gh}\,}}(\beta )=1,\quad {{\,\mathrm{gh}\,}}(\beta ^+)=-2 \end{aligned}$$

In local coordinates we have

56 $$\begin{aligned} S_{\Sigma }(\mathsf {X},\varvec{\eta })=\int _\Sigma \left( \varvec{\eta }_i\wedge {\mathrm {d}}\mathsf {X}^{i}+\frac{1}{2}\pi ^{ij}(\mathsf {X})\varvec{\eta }_i\wedge \varvec{\eta }_j\right) , \end{aligned}$$

where now ${\mathrm {d}}$ denotes the de Rham differential on $\Sigma$. Note that the BV action has the same form as the classical action (49) and thus it produces the same Euler–Lagrange equations, where the classical fields are replaced by the superfields and the de Rham differential ${\mathrm {d}}$ on $\Sigma$ is replaced by the superdifferential $\varvec{D}$.

#### Equivariant BV formulation

Consider a Lie algebra $\mathfrak {g}$ acting on $\Sigma$ via a vector field $v_X$ for some $X\in \mathfrak {g}$. Note that the cohomological vector field is given by57where ${\mathrm {d}}_{\mathcal {F}_\Sigma }$ and  are the Hamiltonian vector fields for the Hamiltonians

58 $$\begin{aligned} S_0=\int _\Sigma \langle \varvec{\eta },{\mathrm {d}}\mathsf {X}\rangle \quad \text {and}\quad S_\pi =\frac{1}{2}\int _\Sigma \langle \pi (\mathsf {X}),\varvec{\eta }\wedge \varvec{\eta }\rangle \end{aligned}$$

respectively. Then one can check that the Classical Master Equation $Q_\Sigma (S_\Sigma )=\{S_\Sigma ,S_\Sigma \}=0$ holds. Consider some variable *u* of cohomological degree 2 and define a $\mathfrak {g}$-DG algebra $\mathcal {O}(\mathcal {F}_\Sigma )[u]:=\mathcal {O}(\mathcal {F}_\Sigma )\otimes \text {Sym}(\mathfrak {g}^*)$. We can define the equivariant extension of the BV action in the Cartan model as

59 $$\begin{aligned} S_\Sigma ^{c}=S_\Sigma +uS_{\iota _{v_X}}, \end{aligned}$$

for $X\in \mathfrak {g}$. Choosing a basis $(e_j)$ of $\mathfrak {g}$, we get60where  is the Hamiltonian of  which is the vector field on $\mathcal {F}_\Sigma$ obtained from the vector field $\iota _{v_j}$, such that61is the differential of the Cartan model of equivariant cohomology. Hence $S_\Sigma ^c\in \mathcal {O}_{\text {loc}}(\mathcal {F}_\Sigma )[u]^\mathfrak {g}$. Moreover, the Classical Master Equation extends to the ***equivariant Classical Master Equation***62where $S_{L_{v_j}}$ is the Hamiltonian of the vector field  which is the vector field on $\mathcal {F}_\Sigma$ defined by $L_{v_j}$. The ***equivariant Quantum Master Equation*** is then given by63For the case where $\Sigma =\mathbb {D}$ we have an $S^1$-action and hence we can consider the $S^1$-equivariant theory. For more details on the equivariant BV construction see [7].

### Splitting of the space of fields

We consider a ***symplectic splitting*** of the space of fields into ***residual fields*** (low energy fields) and ***fluctuations*** (high energy fields), which, for the examples considered in this paper, exists by techniques of Hodge theory (see e.g., [13]). We write

64 $$\begin{aligned} \mathcal {F}_\Sigma =\mathcal {M}_1\times \mathcal {M}_2, \end{aligned}$$

where $\mathcal {M}_1$ is the space of residual fields and $\mathcal {M}_2$ the space of fluctuation fields. We want to assume that $\mathcal {M}_1$ is finite-dimensional, which is the case for *BF*-like theories (such as the Poisson Sigma Model). In this case it is always possible to find a split $\Delta =\Delta _1+\Delta _2$, where $\Delta _j$ is a BV Laplacian on $\mathcal {M}_j$, $j=1,2$. Consider a half-density *f* on $\mathcal {F}_\Sigma$. Then for any Lagrangian submanifold $\mathcal {L}\subset \mathcal {M}_2$ we get

65 $$\begin{aligned} \Delta _1\int _\mathcal {L}f=\int _\mathcal {L}\Delta f. \end{aligned}$$

Here $\int _\mathcal {L}$ denotes the ***BV pushforward***, which is defined on half-densities by restricting the half-density to $\mathcal {L}$ which makes it a density and apply the Berezinian integral. Note that the choice of $\mathcal {L}$ is equivalent to gauge-fixing since, assuming the ***Quantum Master Equation*** $\Delta \exp \left( \frac{\mathrm {i}}{\hbar }S_\Sigma \right) =0\Leftrightarrow \{S_\Sigma ,S_\Sigma \}-2\mathrm {i}\hbar \Delta S_\Sigma =0$, we have an invariance of the BV pushforward $\int _\mathcal {L}\exp \left( \frac{\mathrm {i}}{\hbar }S_\Sigma \right)$ under continuous deformation of $\mathcal {L}$ up to $\Delta _1$-exact terms. This is due to the following theorem.

#### Theorem 5.1

[Batalin–Vilkovisky]. The following holds:

- If $$f=\Delta g$$, then $$\int _\mathcal {L}f=0$$,
- If $\Delta f=0$, then $\frac{{\mathrm {d}}}{{\mathrm {d}}t}\int _{\mathcal {L}_t}f=0$, for a continuous family $(\mathcal {L}_t)$ of Lagrangian submanifolds.

If we take $f=\exp \left( \frac{\mathrm {i}}{\hbar }S_\Sigma \right)$, we get that the Quantum Master Equation has to hold for the second point of the theorem. For *BF*-like theories, $\mathcal {F}_\Sigma$ is given as the direct sum of two complexes $\mathcal {C}\oplus \bar{\mathcal {C}}$ endowed with the differentials $\delta$ and $\bar{\delta }$. We want them to be endowed with a nondegenerate pairing $\langle ,\rangle$ of degree $-1$ such that the differentials are related by $\langle B,\delta A\rangle =\langle \bar{\delta } B,A\rangle$ for all $A\in \mathcal {C}$ and $B\in \bar{\mathcal {C}}$. In that case $\mathcal {M}_1$ is given by the cohomology $\mathcal {H}\oplus \bar{\mathcal {H}}$ and $\mathcal {M}_2$ is just a complement in $\mathcal {F}_\Sigma$. For the case of the Poisson Sigma Model with boundary ($\partial \Sigma \not =\varnothing$) such that the boundary is given by the disjoint union of two boundary components $\partial _1\Sigma$ and $\partial _2\Sigma$ we have

66 $$\begin{aligned} \mathcal {F}_\Sigma =\Omega ^\bullet (\Sigma ,\partial _1\Sigma )\otimes T_xM\oplus \Omega ^\bullet (\Sigma ,\partial _2\Sigma )\otimes T^*_xM[1], \end{aligned}$$

for a constant background field $x:\Sigma \longrightarrow M$, and thus

67 $$\begin{aligned} \mathcal {M}_1=H^\bullet (\Sigma ,\partial _1\Sigma )\otimes T_xM\oplus H^\bullet (\Sigma ,\partial _2\Sigma )\otimes T^*_xM[1]. \end{aligned}$$

According to the splitting of the space of fields, we write $\mathsf {X}=\mathsf {x}+\mathscr {X}$ and $\varvec{\eta }=\mathsf {e}+\mathscr {E}$, where $\mathsf {x},\mathsf {e}\in \mathcal {M}_1$ and $\mathscr {X},\mathscr {E}\in \mathcal {M}_2$.

#### Remark 5.2

Note that functions on the shifted tangent bundle $T[1]\Sigma$ are given by the algebra of differential forms $\Omega ^\bullet (\Sigma )$, which indeed allows us to write the space of fields as in (66). Moreover, if we would have a manifold $\Sigma$ with boundary $\partial \Sigma =\partial _1\Sigma \sqcup \partial _2\Sigma$ as mentioned before, we can split the space of fields as $\mathcal {F}_\Sigma =\mathcal {B}\times \mathcal {M}_1\times \mathcal {M}_2$, where $\mathcal {B}$ would denote the leaf space of the symplectic foliation induced by a chosen polarization on the boundary to perform geometric quantization, where we would choose the convenient $\frac{\delta }{\delta \mathbb {E}}$-polarization on $\partial _1\Sigma$ and the opposite $\frac{\delta }{\delta \mathbb {X}}$-polarization on $\partial _2\Sigma$, where $\mathbb {E}$ and $\mathbb {X}$ denote the $\varvec{\eta }$- and $\mathsf {X}$-boundary fields respectively (elements of the leaf space $\mathcal {B}$). Moreover, one can always obtain a symplectic structure on the space of boundary fields by symplectic reduction. Hence, by techniques of geometric quantization, one would obtain a vector space for each boundary component and one can speak of “boundary states” as elements of these spaces. This construction is needed for treating the Poisson Sigma Model in the Hamiltonian approach of the ***BFV formalism*** (space of boundary fields) coupled together to the BV formalism, which is called the ***BV-BFV formalism*** [12–14]. We will not use the BV-BFV construction, since we will only deal with the disk $\mathbb {D}$ with one single boundary component together with the boundary condition $\iota ^*_{\partial \mathbb {D}}\eta =0$.

### The formal global action

Let us consider for a multivector field $\xi _k\in \Gamma (\bigwedge ^{k}TM)$ the local functional^7^

68 $$\begin{aligned} S_\xi (\mathsf {X},\varvec{\eta }):=\frac{1}{k!}\int _\Sigma \xi ^{i_1,\ldots ,i_k}(\mathsf {X})\varvec{\eta }_{i_1}\wedge \cdots \wedge \varvec{\eta }_{i_k}\in \mathcal {O}_{\text {loc}}(\mathcal {F}_\Sigma ). \end{aligned}$$

Note that for any $k\ge 0$ we have $Q_\Sigma (S_\xi )=\{S_\Sigma ,S_\xi \}=0$. In [21] it was shown that the Poisson Sigma Model action can be formally globalized by adding another term to the action, which is given by

69 $$\begin{aligned} \varphi _x^*S_R(\widehat{\mathsf {X}},\widehat{\varvec{\eta }})=\int _\Sigma R^{j}_{i}(x,\widehat{\mathsf {X}})\widehat{\varvec{\eta }}_j\wedge {\mathrm {d}}x^{i}, \end{aligned}$$

where $\widehat{\mathsf {X}}$ and $\widehat{\varvec{\eta }}$ are defined by the following equations

70 $$\begin{aligned} \mathsf {X}=\varphi _x(\widehat{\mathsf {X}}) ,\quad \varvec{\eta }=({\mathrm {d}}\varphi _x)^{*,-1}\widehat{\varvec{\eta }}. \end{aligned}$$

Recall that $x:\Sigma \longrightarrow M$ denotes a constant background field. Denote by $S_0$ the free part of the action, i.e. $S_0:=\int _\Sigma \varvec{\eta }_i\wedge {\mathrm {d}}\mathsf {X}^{i}$. Lifting the Poisson Sigma Model action to the formal construction, we get the ***formal global action***71If we denote by $\pi ^\varphi :=\mathsf {T}\varphi ^*\pi$, we can observe $\mathsf {T}\varphi ^*S_\pi =S_{\pi ^\varphi }$. Note that the de Rham differential in ${\mathrm {d}}\widehat{\mathsf {X}}^{i}$ is on $\Sigma$ and the de Rham differential in ${\mathrm {d}}x^{i}$ is on the moduli space of constant solutions to the Euler–Lagrange equations

72 $$\begin{aligned} \mathcal {M}_{cl}:=\{(X,\eta )\in \mathcal {F}_\Sigma \mid X=x:\Sigma \longrightarrow M \text { constant map, }\eta =0\}\cong M. \end{aligned}$$

#### Remark 5.3

In general, one can consider any type of classical solution of the Poisson Sigma Model for the point of expansion. We choose the moduli space (72) since it makes things much easier.

One can show that (71,113) satisfies the ***differential Classical Master Equation***

73 $$\begin{aligned} {\mathrm {d}}_xS^{\varphi _x}+\frac{1}{2}\{S^{\varphi _x},S^{\varphi _x}\}=0. \end{aligned}$$

For quantization, consider the partition function, given by (71,113)

74 $$\begin{aligned} Z_x=\int _\mathcal {L}\exp \left( \frac{\mathrm {i}}{\hbar }S^{\varphi _x}\right) \end{aligned}$$

for some Lagrangian submanifold $\mathcal {L}\subset \mathcal {M}_2$. The Quantum Master equation is not satisfied in general. It can be shown that if $\pi$ is divergence free (unimodular), the Quantum Master Equation $\Delta \exp \left( \frac{\mathrm {i}}{\hbar }(S_0+S_\pi )\right) =0$ holds. Another case would be if the Euler-characteristic of $\Sigma$ is zero (e.g., the torus). The choice of a unimodular Poisson structure can be seen as a renormalization procedure. One form of renormalization is to impose that there are no tadpoles (short loops), which results in the fact that

75 $$\begin{aligned} \Delta (\mathsf {X}(s)\varvec{\eta }(s))=\sum _j(-1)^{\vert \mathsf {x}^j\vert }\mathsf {x}^j(s)\wedge \mathsf {e}_j(s)=:\psi (s),\quad \forall s\in \Sigma , \end{aligned}$$

where $\Delta$ is the BV Laplacian acting on the coefficients of the residual fields. If we choose a volume form $\Omega$ on *M*, we can define a divergence operator ${{\,\mathrm{\text {div}}\,}}_\Omega$ and thus a ***renormalized BV Laplacian*** by setting (see also Appendix 9.3)

76 $$\begin{aligned} \Delta S_{\xi }=\int _\Sigma \psi ({{\,\mathrm{\text {div}}\,}}_\Omega \xi )^{i_1,\ldots ,i_{k-1}}(\mathsf {X})\varvec{\eta }_{i_1}\wedge \cdots \wedge \varvec{\eta }_{i_{k-1}}. \end{aligned}$$

Note that $\Delta S_\pi =0$ if ${{\,\mathrm{\text {div}}\,}}_\Omega \pi =0$. Since $\Delta S^{\varphi _x}=0$, we get a ***differential*** version of the Quantum Master Equation

77 $$\begin{aligned} {\mathrm {d}}_x Z_x-\mathrm {i}\hbar \Delta Z_x=0. \end{aligned}$$

#### Remark 5.4

As we will see, the formal global action for the Poisson Sigma Model (see Equation (71,113)) has to be extended to an equivariant version such that the $S^1$-action on the disk is taken into account. This can be done by using the methods of [7] and formulate it as in Equation (59).

## Traces and Algebraic Index Theorem

### Algebraic index theorem

Recall that a ***trace map*** on a Poisson manifold $(M,\pi )$ is a linear functional $\text {Tr}$ on compactly supported functions $f,g\in C^\infty _c(M)$ with values in $\mathbb {R}(\!( \hbar )\!)$ such that

78 $$\begin{aligned} \text {Tr}(f\star g)=\text {Tr}(g\star f) \end{aligned}$$

(hence the name “trace"). There is a canonical trace associated to any star product coming from a symplectic manifold $(M,\omega )$ which is described within the local picture. Locally, all deformations are equivalent to the Weyl algebra and on the Weyl algebra there is a canonical trace which is constructed as an integral with respect to the Liouville measure [26]. If we consider functions with support in neighborhoods of any point of *M*, we set the trace equal to this canonical trace restricted to these functions. Let  denote the  ***-genus*** of *M*, which is a characteristic class of the tangent bundle *TM*. One can express it by a de Rham representative as79where *R* denotes the curvature of any connection on *TM*.

#### Theorem 6.1

(Nest–Tsygan [37]). Let $(M,\omega )$ be a compact symplectic manifold and let $\star$ be a star product with characteristic class $\omega _\hbar =-\omega +\hbar \omega _1+\hbar ^2\omega _2+\cdots$. Then the canonical trace associated to $\star$ obeys80

Consider again a Poisson manifold $(M,\pi )$. Let $A_\hbar :=(C^\infty (M)[\![ \hbar ]\!],\star )$ with star product coming from the Poisson structure $\pi$ (e.g., Kontsevich’s star product), and denote by $CH_\bullet (A_\hbar )$ the ***cyclic homology*** and by $PH_\bullet (A_\hbar )$ the ***periodic cyclic homology***. One can show that $CH_0(A_\hbar )\cong HH_0(A_\hbar )$, where $HH_\bullet (A_\hbar )$ denotes the ***Hochschild homology***. Moreover, as shown by Shoikhet and Dolgushev, the zeroth Hochschild homology is isomorphic to the zeroth ***Poisson homology***^8^$HP_0(M)$. If we assume that there is a volume form $\Omega$ on *M* and that the Poisson structure $\pi$ is unimodular and ${{\,\mathrm{\text {div}}\,}}_\Omega \pi =0$, we can construct a map

81 $$\begin{aligned} \begin{aligned} HP_0(M)&\longrightarrow \mathbb {R},\\ f&\longmapsto \int _Mf\Omega . \end{aligned} \end{aligned}$$

Now we can define an integration map on the zeroth periodic cyclic homology by composition

82 $$\begin{aligned} I:PH_0(A_\hbar )\longrightarrow CH_0(A_\hbar )\xrightarrow {T_{\hbar \pi }} HP_0(M)\xrightarrow {\int _M(-)\Omega } \mathbb {R}, \end{aligned}$$

where we have used Shoikhet’s $T_{\hbar \pi }$ map for the isomorphism $CH_0(A_\hbar )\cong HP_0(M)$ (we could have also used the trace density map of Dolgushev–Rubtsov [25]).

Let $\mathcal {R}$ be a DG ring with differential ${\mathrm {d}}_\mathcal {R}$. For a projective $\mathcal {R}$-module $\mathcal {M}$, one defines a connection to be a map

83 $$\begin{aligned} \nabla :\mathcal {M}\longrightarrow \mathcal {M}\otimes _\mathcal {R}\Omega ^1_\mathcal {R}, \end{aligned}$$

where $\Omega ^1_\mathcal {R}:=\mathcal {R}\otimes \mathcal {R}[1]$, with the usual property

84 $$\begin{aligned} \nabla (r\otimes m)={\mathrm {d}}_{\mathcal {R}}r\otimes m+(-1)^{\vert r\vert }r\nabla m,\quad \forall r\in \mathcal {R},\,m\in \mathcal {M}. \end{aligned}$$

The ***Atiyah class*** of a connection $\nabla$ is then defined by

85 $$\begin{aligned} \mathrm {At}(\nabla ):=[\nabla ,{\mathrm {d}}_\mathcal {R}]\in \Omega ^1_\mathcal {R}\otimes {{\,\mathrm{End}\,}}_\mathcal {R}(\mathcal {M}). \end{aligned}$$

In fact, $[\mathrm {At}(\nabla )]$ measures the the obstruction to find a ${\mathrm {d}}_\mathcal {R}$-compatible connection. We define the ***Chern character*** of a connection $\nabla$ by

86 $$\begin{aligned} \mathrm {Ch}(\nabla ):={{\,\mathrm{Tr}\,}}\exp \left( -\frac{1}{2\pi \mathrm {i}}\mathrm {At}(\nabla )\right) . \end{aligned}$$

Moreover, one can then define more generally the -genus of a connection $\nabla$ on $\mathcal {M}$ in terms of these classes by87where $\mathrm {Td}$ denotes the ***Todd class***, defined by

88 $$\begin{aligned} \mathrm {Td}(\nabla ):=\frac{\mathrm {Ch}(\nabla )}{1-\exp (\mathrm {Ch}(\nabla ))}. \end{aligned}$$

#### Theorem 6.2

(Tamarkin–Tsygan [41]). Let *M* be a compact manifold with formal Poisson structure $\pi \in \hbar \Gamma (\bigwedge ^2TM)[\![ \hbar ]\!]$ and $\Omega$ a volume form on *M* with ${{\,\mathrm{\text {div}}\,}}_\Omega \pi =0$ and $c\in PC_0(A_\hbar )$. Then89Here90where  are the components of the -genus.

### A trace map for negative cyclic chains

In [16] it was shown how one can obtain a trace map by constructing an $L_\infty$-morphism from negative cyclic chains to multivector fields with an adjunction of the formal parameter *u* of degree 2. Moreover, the relation to the BV formulation of the Poisson Sigma Model and how the former formula can be interpreted as an expectation value with respect to the corresponding quantum field theory was shown. However, this construction was only given for open subsets *M* of $\mathbb {R}^d$. We will extend this construction to a global one using notions of formal geometry as we have seen before.

#### Theorem 6.3

[Cattaneo–Felder [16]]. Let *M* be an open subset of $\mathbb {R}^d$ and consider a volume form $\Omega$ on *M*. Denote by $\delta _\Omega :=u{{\,\mathrm{\text {div}}\,}}_\Omega$. Let $A=C^\infty (M)$ and let $(\mathcal {T}^{-\bullet }_{poly}(M),{{\,\mathrm{\text {div}}\,}}_\Omega )$ be the DG module over the DGLA $(\mathcal {T}^{\bullet }_{poly}(M)[u],\delta _\Omega )$ with trivial $(\mathcal {T}^{\bullet }_{poly}(M)[u],\delta _\Omega )$-action. Then there exists an $\mathbb {R}[u]$-linear morphism of $L_\infty$-modules over $(\mathcal {T}^{\bullet }_{poly}(M)[u],\delta _\Omega )$

91 $$\begin{aligned} \mathcal {V}:(CC_{-\bullet }^-(A),b+uB)\longrightarrow (\mathcal {T}^{-\bullet }_{poly}(M)[u],u{{\,\mathrm{\text {div}}\,}}_\Omega ), \end{aligned}$$

such that

1. The zeroth Taylor component $\mathcal {V}_0$ of $\mathcal {V}$ vanishes on $CC^-_m(A)$, $m>0$ and for $f\in A\subset CC_0^-(A)$, $\mathcal {V}_0(f)=f$.
2. For $$\xi \in \Gamma (\bigwedge ^kTM)$$, $$\ell \ge 0$$, $$a=[a_0\otimes \cdots \otimes a_m]\in CC_m^-(A)$$,
  92 $$\begin{aligned} \mathcal {V}_1(\xi u^\ell \mid a)={\left\{ \begin{array}{ll}(-1)^mu^s\xi \blacktriangleleft H(a),&{} \hbox {if} k\ge m \hbox {and} s=k+\ell -m-1\ge 0\\ 0,&{}\text {otherwise}\end{array}\right. } \end{aligned}$$
  where $\blacktriangleleft :\mathcal {T}_{poly}^k(M)\otimes \Omega ^m(M,\mathbb {R})\longrightarrow \mathcal {T}_{poly}^{k-m}(M)$ and *H* is the HKR map.
3. The maps $\mathcal {V}_n$ are equivariant under linear coordinate transformations and^9^
  93 $$\begin{aligned} \mathcal {V}_n(\xi _1\cdots \xi _n\mid a)=\xi _1\wedge \mathcal {V}_{n-1}(\xi _2\cdots \xi _n\mid a) \end{aligned}$$
  whenever $\xi _1=\sum (c^{i}_kx_k+d^{i})\partial _i\in \mathcal {T}^\bullet _{poly}(M)\subset (\mathcal {T}_{poly}^\bullet (M)[u],\delta _\Omega )$ is an affine vector field and $\xi _1,\ldots ,\xi _n\in (\mathcal {T}_{poly}^\bullet (M)[u],\delta _\Omega )$.

The Taylor components of $\mathcal {V}$ are given by maps

94 $$\begin{aligned} \mathcal {V}_n:(\text {Sym}^n\mathcal {T}^{\bullet +1}_{poly}(M)[u],\delta _\Omega )\otimes CC^-_{-\bullet }(A)\longrightarrow \mathcal {T}^{n-1}_{poly}(M). \end{aligned}$$

Note that an element of degree +1 in $(\mathcal {T}_{poly}^\bullet (M)[u],\delta _\Omega )$ has the form $\tilde{\pi }=\pi +uh$, where $\pi$ is a bivector field and *h* a function. The Maurer–Cartan equation $\delta _\Omega \tilde{\pi }-\frac{1}{2}[\tilde{\pi },\tilde{\pi }]=0$ translates to $[\pi ,\pi ]=0$ and

95 $$\begin{aligned} {{\,\mathrm{\text {div}}\,}}_\Omega \pi -[h,\pi ]=0, \end{aligned}$$

and hence $\pi$ is Poisson and *h* corresponds to the Hamiltonian function of the Hamiltonian vector field $\delta _\Omega \pi$. As we have seen, this is equivalent to the unimodularity condition.

#### Remark 6.4

The morphism $\mathcal {V}$ is in fact related to Shoikhets morphism [40] in the proof of Tsygan’s formality theorem on chains [42] for $M=\mathbb {R}^d$. It is a morphism of $L_\infty$-modules over $\mathcal {T}_{poly}^{\bullet +1}(M)$ from $C_\bullet (A)$ to the DG module of differential forms $(\Omega ^{-\bullet }(M,\mathbb {R}),{\mathrm {d}}=0)$ and extends to (11). The action of $\xi \in \mathcal {T}_{poly}^{\bullet +1}(M)$ on $\Omega ^{\bullet }(M,\mathbb {R})$ is given by Lie derivative $L_\xi ={\mathrm {d}}\circ \iota _\xi \pm \iota _\xi \circ {\mathrm {d}}$, where the internal multiplication of vector fields is extended to multivector fields by $\iota _\xi \iota _\zeta =\iota _{\xi \wedge \zeta }$. This construction was globalized by Dolgushev to any manifold *M*. Moreover, recall that a volume form $\Omega \in \Omega ^{d}(M,\mathbb {R})$ defines an isomorphism

96 $$\begin{aligned} \begin{aligned} \mathcal {T}_{poly}^k(M)&\longrightarrow \Omega ^{d-k}(M)\\ \xi&\longmapsto \iota _\xi \Omega , \end{aligned} \end{aligned}$$

and thus we identify the differential ${\mathrm {d}}$ on $\Omega ^\bullet (M,\mathbb {R})$ by the divergence operator ${{\,\mathrm{\text {div}}\,}}_\Omega$ on $\mathcal {T}^{\bullet }_{poly}(M)$. By the fact that $\mathcal {V}$ is an $L_\infty$-morphism we get $\iota _{{{\,\mathrm{\text {div}}\,}}\xi }\Omega ={\mathrm {d}}\iota _\xi \Omega$.

Let

97 $$\begin{aligned} \tilde{\pi }_\hbar :=\hbar \pi +uh, \end{aligned}$$

which is a Maurer–Cartan element if $\pi +uh$ is a Maurer–Cartan element in $(\mathcal {T}_{poly}^\bullet (M)[u][\![ \hbar ]\!],\delta _\Omega )$. We will denote the twist of $\mathcal {V}$ by $\tilde{\pi }_\hbar$ by $\mathcal {V}^{\tilde{\pi }_\hbar }$, which is defined through its Taylor components

98 $$\begin{aligned} \mathcal {V}_n(\tilde{\pi }_\hbar ,\ldots , \tilde{\pi }_\hbar \mid ). \end{aligned}$$

Then we can define a ***trace map*** [16]

99 $$\begin{aligned} \begin{aligned} \text {Tr}^\mathcal {V}:C^\infty _c(M)[\![h]\!]&\longrightarrow \mathbb {R}(\!( \hbar )\!)\\ f&\longmapsto \text {Tr}^\mathcal {V}(f)=\int _M\sum _{n=0}^\infty \frac{1}{n!}\mathcal {V}_n(\tilde{\pi }_\hbar \cdots \tilde{\pi }_\hbar \mid f)\Omega , \end{aligned} \end{aligned}$$

since $\mathcal {V}^{\tilde{\pi }_\hbar }:(CC^-_{-\bullet }(A_\hbar ),b+uB)\longrightarrow (\mathcal {T}_{poly}^{-\bullet }(M)[u][\![ \hbar ]\!],u{{\,\mathrm{\text {div}}\,}}_\Omega )$ is a chain map. We will elaborate on this fact a bit more in Sect. 8.

#### Remark 6.5

For a *d*-manifold *M* denote by $V\mathcal {T}_{poly}^\bullet (M):=\Omega ^d(M,\bigwedge ^\bullet TM)$ differential forms of degree *d* with values in multivector fields. By the isomorphism as mentioned in Remark 6.4, we can construct a natural non-degenerate pairing by

100 $$\begin{aligned} \begin{aligned} \langle ,\rangle :V\mathcal {T}_{poly}^\bullet (M)\otimes \Omega _c^\bullet (M,\mathbb {R})&\longrightarrow \mathbb {R}\\ \xi \Omega \otimes \alpha&\longmapsto \langle \xi \Omega ,\alpha \rangle := \int _M(\iota _\xi \alpha )\Omega , \end{aligned} \end{aligned}$$

where $\xi$ is a $\bullet$-vector field and $\alpha$ is a $\bullet$-form. Here $\Omega$ denotes again a chosen volume form on *M*. We have denoted by $\Omega ^\bullet _c(M,\mathbb {R})$ differential forms with compact support. It is obvious that this map can be extended *u*-bilinearly. Moreover, there is an isomorphism

101 $$\begin{aligned} \begin{aligned} \mathcal {T}_{poly}^\bullet (M)[u]&\longrightarrow V\mathcal {T}_{poly}^\bullet (M)[u]\\ \xi&\longmapsto \xi \otimes \Omega . \end{aligned} \end{aligned}$$

### Construction via the Poisson sigma model

Consider now the Poisson Sigma Model on the disk . Let

102 $$\begin{aligned} Z_0:=\int _\mathcal {L}\exp \left( \frac{\mathrm {i}}{\hbar }S_0\right) , \end{aligned}$$

and define the ***vacuum expectation value*** of an observable by the map

103 $$\begin{aligned} \begin{aligned} \langle \rangle _0:A_\hbar&\longrightarrow \mathbb {R}(\!( \hbar )\!)\\ f&\longmapsto \langle f\rangle _0:=\frac{1}{Z_0}\int _\mathcal {L}\exp \left( \frac{\mathrm {i}}{\hbar }S_0\right) f. \end{aligned} \end{aligned}$$

The map $\mathcal {V}_n$ can be expressed as the vacuum expectation of an observable $S_{\xi _1}\cdots S_{\xi _j}O_{a_0,\ldots ,a_m}$, where

104 $$\begin{aligned} O_{a_0,\ldots ,a_m}:=a_0(\mathsf {X}(t_0))\int _{t_1<t_2<\cdots <t_m\in \partial \mathbb {D}\setminus \{t_0\}}a_1(\mathsf {X}(t_1))\cdots a_m(\mathsf {X}(t_m)). \end{aligned}$$

For *m* points $t_1,\ldots ,t_m\in \partial \mathbb {D}$ we consider the ordering $t_0<\ldots <t_m$, which means that if we start at $t_1$ and move counterclockwise on $\partial \mathbb {D}$, we wil first meet $t_2$, then $t_3$, and so on. If we embed the disk into the complex plane, i.e. we have $\mathbb {D}=\{z\in \mathbb {C}\mid \vert z\vert \le 1\}$ and set $t_0=1$, we can express the counterclockwise condition on $\partial \mathbb {D}$ by $0<\arg (t_1)<\arg (t_2)<\cdots<\arg (t_m)<2\pi$. The cohomology $H^\bullet (\mathbb {D})$ is 1-dimensional and concentrated in degree zero, while the relative cohomology $H^\bullet (\mathbb {D},\partial \mathbb {D})$ is 1-dimensional and concentrated in degree two. So, for $\mathcal {M}_1,\mathcal {H},\bar{\mathcal {H}}$ as defined in Sect. 5.3, we get $\mathcal {H}=(\mathbb {R}^d)^*[-1]$ and $\bar{\mathcal {H}}=\mathbb {R}^d$, thus $\mathcal {M}_1=T^*[-1]M$. Note that functions on $\mathcal {M}_1$ are then multivector fields on *M* with reversed degree and $\Delta _1$ is given by the divergence operator ${{\,\mathrm{\text {div}}\,}}_\Omega$ for the constant volume form. Note that $\Delta _1$ is an operator of degree $+1$. For a function $f\in C^\infty (M)$ and some Lagrangian submanifold $\mathcal {L}\subset \mathcal {M}_2$, we have a map

105 $$\begin{aligned} \text {tr}(f):=\frac{1}{Z_0}\int _\mathcal {L}\exp \left( \frac{\mathrm {i}}{\hbar }(S_0+S_{\tilde{\pi }})\right) O_f=\left\langle \exp \left( \frac{\mathrm {i}}{\hbar }S_{\tilde{\pi }}\right) O_f\right\rangle _0, \end{aligned}$$

given by the expectation value of the corresponding observable. Recall that $\tilde{\pi }$ is a Maurer–Cartan element for a unimodular Poisson structure and that we work with the boundary condition $\iota ^*_{\partial \mathbb {D}}\varvec{\eta }=0$. For two functions $f,g\in C^\infty (M)$, we define $O_f(\mathsf {X},\varvec{\eta }):=f(\mathsf {X}(1))$ for $1\in \partial \mathbb {D}$ and $O_g(\mathsf {X},\varvec{\eta }):=g(\mathsf {X}(0))$ for $0\in \partial \mathbb {D}$. Moreover, define^10^

106 $$\begin{aligned} \text {tr}_2(f,g):=\left\langle \exp \left( \frac{\mathrm {i}}{\hbar }S_{\tilde{\pi }}\right) O_{f,g}\right\rangle _0=\left\langle \exp \left( \frac{\mathrm {i}}{\hbar }S_{\tilde{\pi }}\right) f(\mathsf {X}(t))\int _{s\in \partial \mathbb {D}\setminus \{t\}}g(\mathsf {X}(s))\right\rangle _0, \end{aligned}$$

Then we observe

107 $$\begin{aligned} \Delta _1\text {tr}_2(f,g)=\left\langle \exp \left( \frac{\mathrm {i}}{\hbar }S_{\tilde{\pi }}\right) \delta O_{f,g}\right\rangle _0, \end{aligned}$$

where $\delta$ was the differential on the complex $\mathcal {C}$ in the definition of the space of fields in Sect. 5.3. This follows from the ***Ward identity***

108 $$\begin{aligned} \Delta _1\langle O\rangle _0=\left\langle \Delta O-\frac{\mathrm {i}}{\hbar }\delta O\right\rangle _0, \end{aligned}$$

which is true by (65), the fact that $Z_0$ is constant on $\mathcal {M}_1$ and the Leibniz rule for the BV Laplacian

109 $$\begin{aligned} \Delta (fg)=\Delta (f)g+(-1)^{\vert f\vert }f\Delta (g)-(-1)^{\vert f\vert }\{f,g\},\quad \forall f,g\in C^\infty (M) \end{aligned}$$

(see also Appendix 9.3, Equation (189)). Hence by (51) the two functions *f*, *g* can move under the trace map from both sides^11^ to each other on $\partial \mathbb {D}$. Thus we get

110 $$\begin{aligned} \Delta _1\text {tr}_2(f,g)=\text {tr}(f\star g)-\text {tr}(g\star f). \end{aligned}$$

Hence we get a trace on $C^\infty _c(M)$ by

111 $$\begin{aligned} \text {Tr}^\mathbb {D}(f):=\int _M\text {tr}(f)\Omega . \end{aligned}$$

To globalize the construction, we want to consider the formal global action $S^{\varphi }$ and additional vertices in the ***Feynman graph expansion***. In fact we will have two types of vertices in the bulk, the ones representing the formally lifted Poisson structure $\pi ^\varphi _\hbar :=\mathsf {T}\varphi ^*\tilde{\pi }_\hbar =\hbar \mathsf {T}\varphi ^*\pi -u\mathsf {T}\varphi ^*h$ and the ones representing the *R* vector field coming from the definition of the Grothendick connection. We will also consider additional vertices on the boundary where we place solutions $\gamma$ of (42). Then we can consider the vacuum expectation value

112 $$\begin{aligned} \left\langle \exp \left( \frac{\mathrm {i}}{\hbar }S^\varphi _{\pi ,R}\right) O_{\rho (\mathsf {T}\varphi ^*f)}\right\rangle _0, \end{aligned}$$

where $$S^\varphi _{\pi ,R}:=S_{\pi ^\varphi _\hbar }+\varphi ^*S_R$$.

#### Remark 6.6

The additional vertices labeled by a solution $\gamma$ of (42) give rise to another additional term in the formal global action [21]. In particular, we have to consider the action

113 $$\begin{aligned} \tilde{S}^\varphi =\varphi ^*S_0+S^\varphi _{\pi ,R}+\int _{\partial \mathbb {D}}\widehat{\mathsf {X}}^*\gamma . \end{aligned}$$

We will call the Poisson Sigma Model with action $\tilde{S}^\varphi$ the ***Fedosov-type formal global Poisson Sigma Model*** and we call $\tilde{S}^{\varphi }$ the ***Fedosov-type formal global action***.

#### Proposition 6.7

The map

114 $$\begin{aligned} {{\,\mathrm{Tr}\,}}^\mathbb {D}_\gamma :f\longmapsto \int _M \left\langle \exp \left( \frac{\mathrm {i}}{\hbar }S^\varphi _{\pi ,R}+\frac{\mathrm {i}}{\hbar }\int _{\partial \mathbb {D}}\widehat{\mathsf {X}}^*\gamma \right) O_{\rho (\mathsf {T}\varphi ^*f)}\right\rangle _0\Omega . \end{aligned}$$

coincides with

115 $$\begin{aligned} {{\,\mathrm{Tr}\,}}^\mathcal {V}:f\longmapsto \sum _{n=0}^\infty \frac{1}{n!}\int _M\mathcal {V}_n^{\pi ^\varphi _\hbar }(R\cdots R\mid \rho (\mathsf {T}\varphi ^*f))\Omega , \end{aligned}$$

where we consider $\mathcal {V}^{\pi ^\varphi _\hbar }_n(R\cdots R\mid )$ to be defined on the negative cyclic complex for sections of the Weyl algebra $\mathcal {W}$.

This can be seen by constructing the maps $\mathcal {V}_n$ in terms of graphs. We will do this in Sect. 7.1.

#### Remark 6.8

**Fig. 2:**
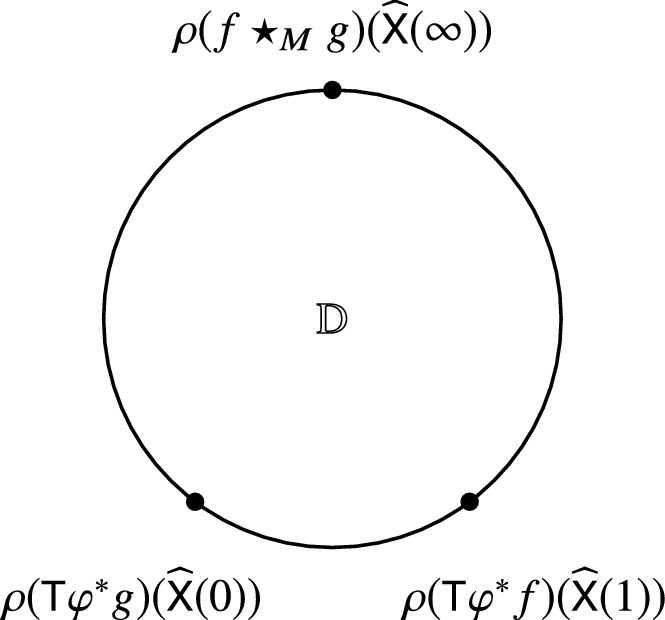
Cyclically ordered points on $S^1=\partial \mathbb {D}$

In fact, one can construct Kontsevich’s star product directly by using a path integral quantization with respect to the formal global action $S^\varphi$ as in (71,113), using a similar approach as in [11], with the difference that the observables on the boundary are given by $\bar{\mathcal {D}}$-closed sections of the form $O_{\rho (\mathsf {T}\varphi ^*f)}$ (see Fig. 2). Hence we can write it down as a path integral

116 $$\begin{aligned} f\star _M g(x)=\rho ^{-1}\left( \int _{\widehat{\mathsf {X}}(\infty )=x} \rho (\mathsf {T}\varphi _x^*f)(\widehat{\mathsf {X}}(1))\rho (\mathsf {T}\varphi _x^*g)(\widehat{\mathsf {X}}(0))\exp \left( {\frac{\mathrm {i}}{\hbar }S^{\varphi _x}(\widehat{\mathsf {X}},\widehat{\varvec{\eta }})}\right) \right) \Bigg |_{y=0}\nonumber \\ \end{aligned}$$

## Feynman Graphs for the Globalized Action

### Construction via graphs

We want to describe how the Taylor components of $\mathcal {V}$ are given in terms of graphs. In fact we have

117 $$\begin{aligned} \mathcal {V}_n(\xi \mid a)=\sum _{\Gamma \in \mathcal {G}_{\mathbf {k},m}}w_\Gamma \mathcal {V}_\Gamma (\xi \mid a), \end{aligned}$$

where $\xi =\xi _1 \cdots \xi _n$, with $\xi _i\in \Gamma (\bigwedge ^{k_i}TM)[u]$, $\mathbf {k}=(k_1,\ldots ,k_n)$ and $a=[a_0\otimes \cdots \otimes a_m]\in C_m(A)$. Here $w_\Gamma \in \mathbb {R}$ denotes the weight of a graph $\Gamma$ according to the given Feynman rules, which can be computed as integrals over configuration spaces of points on the the interior of the disk and on the boundary. We want to recall the definition of the finite set $\mathcal {G}_{\mathbf {k},m}$ of oriented graphs as in [16].

For each graph^12^$\Gamma \in \mathcal {G}_{\mathbf {k},m}$ with $n+m$ vertices (*n* vertices in the bulk and *m* vertices on the boundary), we assign a vertex set $V(\Gamma )=V_1(\Gamma )\sqcup V_2(\Gamma )\sqcup V_w(\Gamma )$. We will distinguish between two different types of vertices which we call the ***black vertices*** $V_b(\Gamma )=V_1(\Gamma )\sqcup V_2(\Gamma )$ and the ***white vertices*** $V_w(\Gamma )$. Within the black vertices we will also distinguish between vertices of ***type 1*** and of ***type 2*** according to the following rules.

- There are *n* vertices in $V_1(\Gamma )$. There are exactly $k_i$ edges originating at the *i*th vertex of $V_1(\Gamma )$.
- There are *m* vertices in $V_2(\Gamma )$. There are no edges originating at these vertices.
- There is exactly one edge pointing at each vertex in $V_w(\Gamma )$ and no edge originating from it.
- There are no edges starting and ending at the same vertex.
- For each pair of vertices (*i*, *j*) there is at most one edge from *i* to *j*.

Each multivector field $\xi _i$ can be endowed with a power of the formal parameter $v^\ell$, which represent the residual field assigned to a black vertex.

#### Example 7.1

**Fig. 3:**
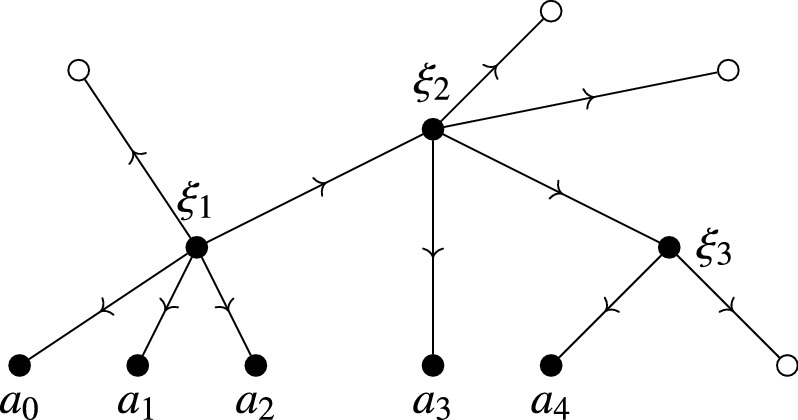
Example of a graph $\Gamma$

Let $\Gamma$ be the graph constructed as in Fig. 3 using the multivector fields $\xi _1,\xi _2,\xi _3\in \Gamma (\bigwedge ^\bullet TM)$ with $\vert \xi _1\vert =5,\vert \xi _2\vert =4$, and $\vert \xi _3\vert =2$. Then we get

118 $$\begin{aligned}&\mathcal {V}_\Gamma (\xi _1u^{\ell _1}\xi _2u^{\ell _2}\xi _3u^{\ell _3}\mid [a_0\otimes a_1\otimes a_2\otimes a_3\otimes a_4])\nonumber \\&\quad = \sum \xi _1^{i_1i_2i_3i_4i_5}\partial _{i_1}\xi _2^{j_1j_2j_3j_4}\partial _{j_1}\xi _3^{m_1m_2}\partial _{i_2}a_0\partial _{i_3}a_1\partial _{i_4}a_2\partial _{j_2}a_3\partial _{m_1}a_4\theta _{i_5}\theta _{j_3}\theta _{j_4}\theta _{m_2},\nonumber \\ \end{aligned}$$

where we sum over all indices and where we set $\theta _i:=\frac{\partial }{\partial x^{i}}$ for local coordinates $(x^i)$ on *M*.

To compute the configuration integrals, we want to make a degree count, i.e. we want the form degree to be equal to the dimension of the configuration space. Let $\Sigma$ be a manifold with boundary and define the ***configuration space*** of *n* points in the bulk and *m* points on the boundary by

119 $$\begin{aligned} \mathsf {Conf}_{n,m}(\Sigma ):=\{(x_1,\ldots ,x_n,y_1,\ldots ,y_m)\in \mathrm {int}(\Sigma ^n)\times (\partial \Sigma )^n\mid x_i\not =x_j,\, y_i\not =y_j\,\forall i\not =j\}.\nonumber \\ \end{aligned}$$

Moreover, denote by $\mathsf {C}_{n,m}(\Sigma )$ the ***FMAS-compactification*** [1, 28] of $\mathsf {Conf}_{n,m}(\Sigma )$ (or of its quotient with respect to the corresponding group action).

Let us give some ideas of the FMAS-comnpactification construction. Let *S* be a finite set and consider the space ${{\,\mathrm{Map}\,}}(S,\Sigma )$ of maps from *S* to $\Sigma$. Moreover, consider the smooth blow up $\mathrm {B}\ell ({{\,\mathrm{Map}\,}}(S,\Sigma ),\Delta _S)$, where $\Delta _S$ denotes the diagonal $\Delta _S\subset {{\,\mathrm{Map}\,}}(S,\Sigma )$, consisting of constant maps $S\rightarrow \Sigma$. Denote by $\mathsf {Conf}_S(\Sigma )$ the space of embeddings of *S* into $\Sigma$. One can then observe that for every inclusion $K\subset S$ there are natural projections ${{\,\mathrm{Map}\,}}(S,\Sigma )\rightarrow {{\,\mathrm{Map}\,}}(K,\Sigma )$ and corresponding arrows $\mathsf {Conf}_S(\Sigma )\rightarrow \mathsf {Conf}_K(\Sigma )$ by restriction of maps from *S* to *K* as a functorial approach. Further, one can show that the inclusions $\mathsf {Conf}_S(\Sigma )\subset {{\,\mathrm{Map}\,}}(S,\Sigma )$ can be lifted to inclusions $\mathsf {Conf}_S(\Sigma )\subset \mathrm {B}\ell ({{\,\mathrm{Map}\,}}(S,\Sigma ),\Delta _S)$ since these sets avoid all diagonals. Thus, for a finite set *X*, we have a canonical inclusion

$$\begin{aligned} \mathsf {Conf}_X(\Sigma )\hookrightarrow \bigotimes _{S\subset X\atop \vert S\vert \ge 2}\mathrm {B}\ell ({{\,\mathrm{Map}\,}}(S,\Sigma ),\Delta _S)\times {{\,\mathrm{Map}\,}}(S,\Sigma ). \end{aligned}$$

The FMAS-compactification, $\mathsf {C}_X(\Sigma )$, is then defined as the closure of $\mathsf {Conf}_X(\Sigma )$ in this embedding.

Let now $\Sigma =\mathbb {D}$ and fix the point 1 on $\partial \mathbb {D}$. Then we have to work on the section space

120 $$\begin{aligned}&\mathsf {C}^0_{n,m}(\mathbb {D}):=\{(z,t)\in (\mathrm {int}(\mathbb {D}))^n\times (\partial \mathbb {D})^m\mid z_i\not =z_j\,(i\not =j),\,\nonumber \\&\qquad 0<\arg (t_1)<\cdots<\arg (t_m)<2\pi \}. \end{aligned}$$

The space (120) has dimension $2n+m$. Moreover, the number *m* represents the amount of points on the boundary distinct from the fixed point 1, i.e. the total amount of points on the boundary is $m+1$. In fact, (120) is equal to the set $\{(z,t)\in \mathsf {C}_{n,m+1}(\mathbb {D})\mid t_0=1\}$ for $m\ge 1$.

As already mentioned, we have an $S^1$-action on the disk. Instead of working with the quotient of the configuration space by $PSL_2(\mathbb {R})$, we will work with ***equivariant differential forms***, which arise from the equivariant BV construction of the Poisson Sigma Model within the Feynman graph expansion.

### Equivariant differential forms and equivariant Stokes’ theorem

We want to work with equivariant differential forms with respect to the $S^1$-action on the disk. We define them as

121 $$\begin{aligned} \Omega _{S^1}^\bullet (\mathbb {D}):=\Omega ^\bullet (\mathbb {D})^{S^1}[u], \end{aligned}$$

where the differential is given by ${\mathrm {d}}_{S^1}:={\mathrm {d}}-u\iota _\mathbf {v}$. Here $\mathbf {v}\in \Gamma (T\mathbb {D})$ denotes the image of the infinitesimal vector field $\frac{{\mathrm {d}}}{{\mathrm {d}}t}$, which is the generator of the infinitesimal action $\mathbb {R}\frac{{\mathrm {d}}}{{\mathrm {d}}t}\longrightarrow \Gamma (T\mathbb {D})$. Now consider a differential form $\omega$ on the configuration space $\mathsf {C}^0_{n,m}(\mathbb {D})$. We want to describe the boundary of the configuration space. Let *S* be a subset of ${\bar{n}}\ge 2$ points in the bulk which collapse at a point in the bulk of the disk. Then the ***stratum of type I*** is given by

122 $$\begin{aligned} \partial _S\mathsf {C}_{n,m}(\mathbb {D})\cong \mathsf {C}_{{\bar{n}}}(\mathbb {C})\times \mathsf {C}^0_{n-{\bar{n}}+1,m}(\mathbb {D}). \end{aligned}$$

The ***stratum of type II*** is constructed as follows. Let *S* be the subset of ${\bar{n}}$ points in the bulk and *T* the subset of ${\bar{m}}$ points on the boundary which collapse at a point on the boundary of the disk. Hence we get the stratum

123 $$\begin{aligned} \partial _{S,T}\mathsf {C}_{n,m}(\mathbb {D})\cong \mathsf {C}_{{\bar{n}}, {\bar{m}}}(\mathbb {H})\times \mathsf {C}^0_{n-{\bar{n}},m-{\bar{m}}+1}(\mathbb {D}), \end{aligned}$$

where $\mathbb {H}$ denotes the upper half plane.

#### Theorem 7.2

(Equivariant Stokes [16]). Let $\omega \in \Omega ^\bullet _{S^1}(\mathsf {C}_{n,m+1}(\mathbb {D}))$. Denote also by $\omega$ its restriction on $\mathsf {C}^0_{n,m}(\mathbb {D})\subset \mathsf {C}_{n,m+1}(\mathbb {D})$. Denote by $\omega ^\partial$ its restriction to the coboundary 1 strata $\partial _i\mathsf {C}^0_{n,m}(\mathbb {D})$. Then

124 $$\begin{aligned} \int _{\mathsf {C}^0_{n,m}(\mathbb {D})}{\mathrm {d}}_{S^1}\omega =\sum _i\int _{\partial _i\mathsf {C}^0_{n,m}(\mathbb {D})}\omega ^\partial -u \int _{\mathsf {C}_{n,m+1}(\mathbb {D})}\omega \end{aligned}$$

### Weights of graphs

We will consider a ***propagator*** $\mathscr {P}$ on $\mathbb {D}\times \mathbb {D}\setminus \mathsf {diag}$, where $\mathsf {diag}:=\{(z,z)\mid z\in \mathbb {D}\}\subset \mathbb {D}\times \mathbb {D}$ denotes the diagonal on the disk. The propagator will be a 1-form on the configuration space of the disk. In particular, we have

125 $$\begin{aligned} \mathscr {P}(z,w):=\frac{1}{4\pi \mathrm {i}}\left( {\mathrm {d}}\log \frac{(z-w)(1-z{\bar{w}})}{({\bar{z}}-{\bar{w}})(1-{\bar{z}} w)}+z{\mathrm {d}}{\bar{z}}-{\bar{z}}{\mathrm {d}}z\right) . \end{aligned}$$

Note that this propagator is equivariant under the $S^1$-action.

#### Remark 7.3

An important fact [10, 13] of the propagator is

126 $$\begin{aligned} {\mathrm {d}}\mathscr {P}(z_1,z_2)=\pm \sum _j \pi _1^*\chi _j\wedge \pi _2^*\chi ^j=\pm \Delta _1(\mathsf {x}_1\wedge \mathsf {e}_2), \end{aligned}$$

where $\pi _1,\pi _2$ are the projections to the first and second factor respectively. Here $\chi _j,\chi ^j$ are representatives of the cohomology classes and their duals respectively, such that $\int _\mathbb {D}\chi _i\wedge \chi ^j=\delta _i^j$.

Computing this directly, we get

127 $$\begin{aligned} {\mathrm {d}}_{S^1}\mathscr {P}&={\mathrm {d}}\mathscr {P}-u\iota _\mathbf{v }\mathscr {P} \end{aligned}$$

128 $$\begin{aligned}&=\frac{1}{4\pi \mathrm {i}}{\mathrm {d}}(z{\mathrm {d}}{\bar{z}}-{\bar{z}}{\mathrm {d}}z)-u\iota _\mathbf{v }\mathscr {P} \end{aligned}$$

129 $$\begin{aligned}&=-\pi _1^*\left( \frac{\mathrm {i}}{2\pi }{\mathrm {d}}z\wedge {\mathrm {d}}{\bar{z}}+u(1-\vert z\vert ^2)\right) . \end{aligned}$$

The first term of (129) is a volume form on the disk and hence a representative of the cohomology class, hence the whole is a representative of the equivariant cohomology class.

**Fig. 4:**
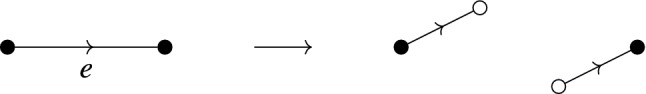
Edge split

Graphically, this corresponds to the fact that if the de Rham differential acts on an edge of a graph between two (black) vertices (which represents a propagator), it will split into residual fields (see Fig. 4). This can be extended to the equivariant differential ${\mathrm {d}}_{S^1}$. The white vertices mentioned in the graph construction before are actually represented by zero modes on $\mathbb {D}$. More precisely, we have the following Lemma.

#### Lemma 7.4

(e.g., [13, 16]). Let $\partial _e\Gamma$ be the graph which is obtained from the graph $\Gamma$ by adding a white vertex $\circ$ and replacing the edge $e\in E_b(\Gamma )$ connecting two black vertices by an edge originating at the same vertex as *e* but ending at the white vertex $\circ$. Then

130 $$\begin{aligned} {\mathrm {d}}_{S^1}\omega _\Gamma =\sum _{e\in E_b(\Gamma )}(-1)^{\vert E_b(\Gamma )\vert }\omega _{\partial _e\Gamma }. \end{aligned}$$

The represented zero modes are parametrized by the formal variable $u^\ell$ attached to each vertex. The weight of a graph $\Gamma \in \mathcal {G}_{(k_1,\ldots ,k_n),m}$ is then computed by

131 $$\begin{aligned} w_\Gamma =\frac{1}{k_1!\cdots k_n!}\int _{\mathsf {C}^0_{n,m}(\mathbb {D})}\omega _\Gamma . \end{aligned}$$

The ***equivariant cohomology*** $H^\bullet _{S^1}(\mathbb {D})$ is generated by the constant function 1. Moreover, the ***relative equivariant*** cohomology $H^\bullet _{S^1}(\mathbb {D},\partial \mathbb {D})$ is generated by the class of

132 $$\begin{aligned} \phi (z,u):=\frac{\mathrm {i}}{2\pi }{\mathrm {d}}z\wedge {\mathrm {d}}{\bar{z}}+u(1-\vert z\vert ^2). \end{aligned}$$

#### Remark 7.5

Note that with this notation we have ${\mathrm {d}}_{S^1}\mathscr {P}=-\pi _1^*\phi$.

The differential form $\omega _\Gamma \in \Omega ^{2n+m}_{S^1}(\mathsf {C}^0_{n,m}(\mathbb {D}))$ is given by

133 $$\begin{aligned} \omega _\Gamma =\bigwedge _{i\in V_1(\Gamma )}\bigwedge _{(i,j)\in E_b(\Gamma )}\mathscr {P}(z_i,z_j)\bigwedge _{i\in V_1(\Gamma )}\phi (z_i,u)^{r_i}, \end{aligned}$$

where the number $r_i$ is given by the degree of the vertex *i* plus the amount of white vertices attached to it. Moreover, we have the following lemma.

#### Lemma 7.6

([16, 36]) For all $z,z'\in \mathbb {D}$ we have

134 $$\begin{aligned} \int _{w\in \mathbb {D}}\mathscr {P}(z,w)\wedge \mathscr {P}(w,z')=0. \end{aligned}$$

Moreover, for all $z\in \mathbb {D}$ we have

135 $$\begin{aligned} \int _{w\in \mathbb {D}}\mathscr {P}(z,w)\wedge \phi (w,u)=0. \end{aligned}$$

**Fig. 5:**
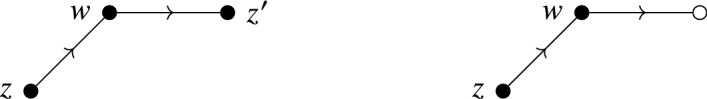
The first picture corresponds to the integrand of (134) and the second picture corresponds to the one of (135). Graphs with such a vertex *w* vanish

## Trace Property and Relation to the Algebraic Index Theorem

### Proof of the trace property

#### Theorem 8.1

The map (115) is a trace on the algebra $(C^\infty _c(M)[\![ \hbar ]\!],\star )$.

#### Proof

This follows by the fact that

136 $$\begin{aligned} \mathcal {V}^{\pi ^\varphi _\hbar }:(CC^-_{-\bullet }(A_\hbar ),b+uB)\longrightarrow (\mathcal {T}^{-\bullet }_{poly}(M)[u][\![\hbar ]\!],u{{\,\mathrm{\text {div}}\,}}_{\Omega }) \end{aligned}$$

is a chain map, which follows from Theorem 7.2 and Lemma 7.4. Using the construction with the Poisson Sigma Model, the trace property follows from (51) and the constructions in Sect. 6.3.

Indeed, consider the observable $O_{\rho (\mathsf {T}\varphi ^*f),\rho (\mathsf {T}\varphi ^*g)}$ for $f,g\in C_c^\infty (M)[\![ \hbar ]\!]$. Note that the configuration integrals are considered on the section space where the point 1 is fixed on the boundary, labeled by the observable $O_{\rho (\mathsf {T}\varphi ^*f)}$. We consider another point 0 on the boundary, which is not fixed, labeled by the observable $O_{\rho (\mathsf {T}\varphi ^*g)}$. Moreover, we have some additional $m-1$ boundary points labeled by $\gamma$. Note that there are boundary strata of the configuration space where *g* collides to *f* from the left and one where it collides from the right. Recall that the dimension of the configuration space $\mathsf {C}^0_{n,m}(\mathbb {D})\subset \mathsf {C}_{n,m+1}(\mathbb {D})$ is given by $2n+m$. Without the point 0 we would have that the dimension is equal to $2n+m-1$, which has to be the same as the form degree of the differential form $\omega _\Gamma$ within the configuration integral for any graph $\Gamma \in \mathcal {G}_{(k_1,\ldots ,k_n),m}$. Hence, we look at its equivariant differential ${\mathrm {d}}_{S^1}\omega _\Gamma$ and apply the equivariant Stokes’ theorem (Theorem 7.2). Using (117) and (131), we can write137where $\mathbf {k}=(k_1,\ldots ,k_n)$ and $\Gamma '<\Gamma$ is a subgraph of $\Gamma$, where $n'<n$ points collapse in the bulk and $m'<m$ collapse on the boundary. Moreover,  is a graph whose vertex set satisfies  with the same amount of vertices in the bulk and on the boundary plus an additional vertex on the boundary. Note that by setting $u=0$, Theorem 7.2 reduces to the usual Stokes’ theorem for corners. The dimension of the configuration space $\mathsf {C}_{n,m}(\mathbb {H})$ modulo scaling and translation is given by $2n+m-2$. This has to be equal to the form degree of the differential form we want to integrate. Let *p* be the amount of vertices labeled by $\pi _\hbar ^\varphi$ and *r* the amount of vertices labeled by *R*. Then we have

138 $$\begin{aligned} \begin{aligned} 2n+m-2&=2p+r,\\ n&=p+r. \end{aligned} \end{aligned}$$

**Fig. 6:**
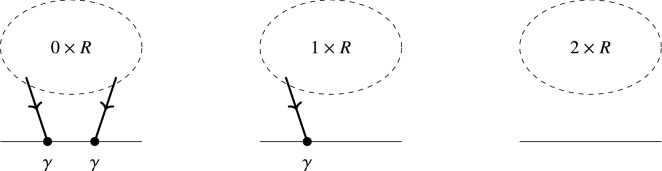
Illustration of the three different cases. The dashed ellipses represent a graph $\Gamma$ in the bulk of the disk with either $r=0$ (first picture), $r=1$ (second picture), or $r=2$ (third picture). Note that in each picture *p* can be arbitrary. The thick arrows denote the fact that there can be arbitrarily many incoming arrows, depending on the combinatorics

This implies three different cases (see Fig. 6);

- $$r=2,m=0$$,
- $$r=m=1$$,
- $$r=0,m=2$$.

Summing over all these graphs, the third picture will exactly correspond to $F=F(R,R)$, the curvature of the deformed Grothendieck connection $\mathcal {D}$, the second picture to $\mathcal {D}\gamma$ and the first picture is exactly the star product $\gamma \star \gamma$. Thus, summing them together we get a contribution

139 $$\begin{aligned} F+\mathcal {D}\gamma +\gamma \star \gamma =0, \end{aligned}$$

and hence these terms vanish. Hence the only strata that survive within the boundary of the configuration space are the ones where *g* approaches *f* from the left and from the right, so by [11] we get the boundary contribution $g\star f-f\star g$.

In fact, for any $\xi =\xi _1\cdots \xi _n\in (\text {Sym}^n\mathcal {T}^{\bullet +1}_{poly}(M)[u],\delta _\Omega )$ and $a\in CC^-_m(A)$, we have

140 $$\begin{aligned}&\mathcal {V}_n(\delta _\Omega \xi \mid a)+(-1)^{\vert \xi \vert +m}\mathcal {V}_n(\xi \mid (b+uB)a)\nonumber \\&\quad +\sum _{k=0}^{n-1}\sum _{\sigma \in S_{k,n-k}}(-1)^{\vert \xi \vert -1}\varepsilon (\sigma ,\xi )\mathcal {V}_k(\xi _{\sigma (1)}\cdots \xi _{\sigma (k)}\mid \mathcal {U}_{n-k}(\bar{\xi }_{\sigma (k+1)}\cdots \bar{\xi }_{\sigma (n)}))\cdot a)\nonumber \\&\quad +\sum _{i<j}\varepsilon _{ij}\mathcal {V}_{n-1}((-1)^{\vert \xi _i\vert -1}[\xi _i,\xi _j]_{SN}\cdot \xi _1\cdots \bar{\xi }_i\cdots \bar{\xi }_j\cdots \xi _n\mid a)={{\,\mathrm{\text {div}}\,}}_\Omega \mathcal {V}_n(\xi \mid a),\nonumber \\ \end{aligned}$$

where $\bar{\xi }_i$ denotes the projection of $\xi _i$ to $\mathcal {T}^{\bullet +1}_{poly}(M)$, $S_{p,q}\subset S_{p+q}$ is the set of (*p*, *q*)-shuffles and the signs $\varepsilon (\sigma ,\xi ),\varepsilon _{ij}$ are the Koszul signs coming from the permutation of the $\xi _i$, and $\vert \xi \vert =\sum _{i}\vert \xi _i\vert$. Note that $\delta _\Omega$ is extended to a degree $+1$ derivation on $\text {Sym}\mathcal {T}^{\bullet +1}_{poly}(M)[u]$. The maps $\mathcal {U}_k:\text {Sym}^k\mathcal {T}^{\bullet +1}_{poly}(M)\longrightarrow \mathcal {D}^{\bullet +1}_{poly}(M)$ are the Taylor components of Kontsevich’s $L_\infty$-morphism.

Indeed, one can show that for any $a=[a_0\otimes \cdots \otimes a_m]\in C_{-m}(A)$, $\Gamma \in \mathcal {G}_{\mathbf {k},m}$ and $\xi =\xi _1\cdots \xi _n$ with $\xi _i\in \Gamma (\bigwedge ^{k_i}TM)$ we have^13^

141 $$\begin{aligned} {{\,\mathrm{\text {div}}\,}}_\Omega \mathcal {V}_n(\xi \mid a)-\mathcal {V}_n(\delta _\Omega \xi \mid a)=\sum _{(\Gamma ,e)}(-1)^{\vert E_b(\Gamma )\vert }w_{\partial _e\Gamma }\mathcal {V}_\Gamma (\xi \mid a), \end{aligned}$$

by identifying $\Gamma (\bigwedge ^n TM)$ with $C^\infty (M)[\theta _1,\ldots ,\theta _n]$, where $\theta _i$ are odd variables such that ${{\,\mathrm{\text {div}}\,}}_\Omega =\sum _{1\le i\le n}\frac{\partial ^2}{\partial t_i\partial \theta _i}$. In fact, we have

142 $$\begin{aligned} \sum _{e\in E_b(\Gamma )}(-1)^{\vert E_b(\Gamma )\vert }w_{\partial _e\Gamma }=\sum _{i}\int _{\partial _i\mathsf {C}^0_{n,m}(\mathbb {D})}\omega ^\partial _\Gamma -u\sum _{k=0}^m(-1)^{km}\int _{\mathsf {C}^0_{n,m+1}(\mathbb {D})}j^*_k\omega _\Gamma ,\nonumber \\ \end{aligned}$$

where $j_k$ is defined as follows: Define a map

143 $$\begin{aligned} \begin{aligned} j_0:\mathsf {C}^0_{n,m}(\mathbb {D})&\longrightarrow \mathsf {C}_{n,m}(\mathbb {D})\\ (z,1,t_1,\ldots ,t_m)&\longmapsto (z,t_1,\ldots ,t_m) \end{aligned} \end{aligned}$$

Moreover, define a map

144 $$\begin{aligned} \begin{aligned} \lambda :\mathsf {C}^0_{n,m}(\mathbb {D})&\longrightarrow \mathsf {C}^0_{n,m}(\mathbb {D})\\ (z_1,\ldots ,z_n,1,t_1,\ldots ,t_m)&\longmapsto (z_1,\ldots ,z_n,1,t_m,t_1,\ldots ,t_{m-1}) \end{aligned} \end{aligned}$$

Then the collection $j_k:= j_0\circ \underbrace{\lambda \circ \cdots \circ \lambda }_{k \text { times}}$, for $k=0,1,\ldots ,m-1$ defines an embedding

145 $$\begin{aligned} j:\mathsf {C}^0_{n,m}(\mathbb {D})\sqcup \cdots \sqcup \mathsf {C}^0_{n,m}(\mathbb {D})\mathsf {C}_{n,m}(\mathbb {D}). \end{aligned}$$

Moreover, note that

146 $$\begin{aligned} \int _{\mathsf {C}_{n,m+1}(\mathbb {D})}\omega =\sum _{k=0}^m(-1)^{km}\int _{\mathsf {C}^0_{n,m+1}(\mathbb {D})}j^*_k\omega , \end{aligned}$$

and the second term on the right-hand side of (142) is given by $\mathcal {V}_{n+1}(\xi \mid Ba)$. Let us look at the boundary integral in the first term of the right hand side of (142). As argued in [16], one can show that treating the boundary strata of type I, the only remaining term will be the sum in (140) containing the Schouten–Nijenhuis bracket. The strata of type II will give a contribution as the sum in (140) containing Kontsevich’s $L_\infty$-morphism and a term $\mathcal {V}_{n-1}(\xi \mid ba)$.

Note that (142) together with (110), (126) and Lemma 7.4 ensure that ${{\,\mathrm{Tr}\,}}^\mathcal {V}(f\star g)={{\,\mathrm{Tr}\,}}^\mathcal {V}(g\star f)$ since ${{\,\mathrm{\text {div}}\,}}_\Omega \pi ^\varphi _\hbar =0$.

Using Equation (140), we get that the twist of $\mathcal {V}$ by $\pi ^\varphi _\hbar$ is indeed a chain map. Recall from Sect. 6 that the zeroth cyclic homology $CH_0(A_\hbar )$ is isomorphic to the zeroth Hochschild homology $HH_0(A_\hbar )$, which is again isomorphic to the zeroth Poisson homology $HP_0(M)$. Hence the chain map $\mathcal {V}^{\pi ^\varphi _\hbar }$ induces a map

147 $$\begin{aligned} C^\infty (M)[\![\hbar ]\!][u,u^{-1}]\cong CH_0(A_\hbar )\cong HH_0(A_\hbar )\xrightarrow {\mathcal {V}^{\pi ^\varphi _\hbar }} HP_0(M)\longrightarrow \mathbb {R}(\!(\hbar )\!)[u,u^{-1}],\nonumber \\ \end{aligned}$$

given by integration as in (81). $\quad \square$

### Relation to the Tamarkin–Tsygan theorem

#### Theorem 8.2

The trace formula in (115) evaluated at any periodic cyclic chain $c\in PC_{-m}(A_\hbar )$ is given by (89).

#### Proof

Note that since the Lie derivative with respect to the Poisson tensor $\pi$ is defined by $L_\pi :={\mathrm {d}}\circ \iota _\pi -\iota _\pi \circ {\mathrm {d}}$, we get an isomorphism of complexes

148 $$\begin{aligned} \begin{aligned} (\Omega ^{-\bullet }(M,\mathbb {R})[\![ \hbar ]\!][u,u^{-1}],L_\pi +u{\mathrm {d}})&\longrightarrow (\Omega ^{-\bullet }(M,\mathbb {R})[\![ \hbar ]\!][u.u^{-1}],u{\mathrm {d}})\\ \alpha&\longmapsto \alpha \exp \left( \iota _\pi /u\right) . \end{aligned} \end{aligned}$$

**Fig. 7:**
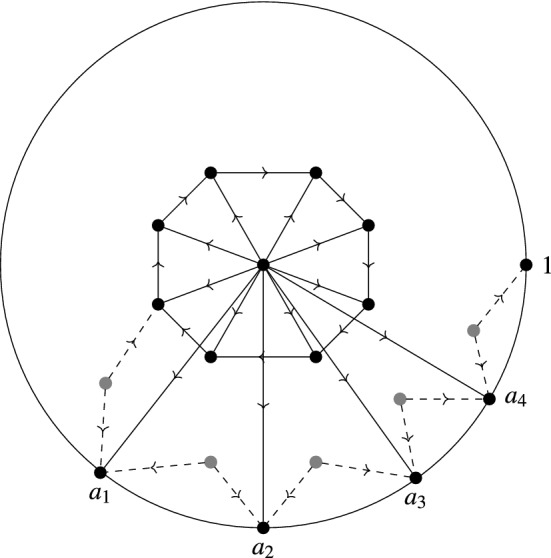
Illustration of the merging of all $\pi _\hbar ^\varphi$-vertices (illustrated in gray) to the center of the disk. The black vertices on the wheel contain the curvature coming from the Grothendieck connection by the 1-forms *R*

Let $A_t$ be a 1-parameter family of algebras given by $A[\![t]\!]$ as an $\mathbb {R}[t]$-module. Denote by $\mathcal {V}^\pi :=\sum _{n=0}^\infty \frac{1}{n!}\mathcal {V}_n^\pi$. Consider the ***Gauss–Manin connection*** on the periodic cyclic cohomology viewed as a vector bundle over the parameter space (see e.g [20, 32, 43]). In [20] it was shown that for a 1-parameter family $\pi _t$ of solutions to the Maurer–Cartan equation e.g., with polynomial dependence $\pi _t=t\pi$ for a Poisson tensor $\pi$, and a cyclic cycle $c_t\in PC_{-m}(A_t)$, which is horizontal with respect to the Gauss–Manin connection, the class of $((\exp \left( \iota _{\pi _t}/u\right) \mathcal {V}^{\pi _t})(c_t)$ in $\bigoplus _{j\ge 0}H^{m+2j}(M,\mathbb {R})[\![ \hbar ]\!]u^j$ is independent of *t*. Denote by  the image of $\mathcal {V}^{\pi _t}$ under the isomorphism (148) with formal Poisson structure $\pi _t$. Since  is independent of *t*, we can set the Poisson tensor to be zero. In our case we have $t=\hbar$ and this will allow us to put $\hbar =0$ in $\pi ^\varphi _\hbar$ and to merge all the $\pi$ vertices at zero (see Fig. 7). This will produce wheel graphs as considered e.g., in [44, 45]. Let us denote the weight for a wheel graph with *j* vertices by $w_j$.

Note that the curvature of the Grothendieck connection is contained in the *R*-vertices (see Sect. 4.5). Hence, considering the Propagator $\mathscr {P}$ on the disk we can compute the weight of a wheel diagram with *j* black vertices. We will get

149 $$\begin{aligned} w_{j}=\int _{\mathsf {C}^0_{j+1,0}(\mathbb {D})}\mathscr {P}(z_1,z_2)\wedge \cdots \wedge \mathscr {P}(z_j,z_1)\wedge \bigwedge _{k=1}^{j}\mathscr {P}(0,z_k) \end{aligned}$$

where $z_1,\ldots ,z_j$ are the vertices labeled by *R*. Moreover, if we recall that $R_\ell (x,y)=R^{k}_\ell (x,y)\frac{\partial }{\partial y^k}$ and $R^k(x,y):=R^k_\ell (x,y) {\mathrm {d}}x^\ell$, we get a differential form

150 $$\begin{aligned} \mathcal {V}_j(R\cdots R\mid 1)=\sum \partial _{\ell _1}\partial _{k_j}R^{k_1}_{\ell _1}(x,y){\mathrm {d}}x^{\ell _1}\wedge \partial _{\ell _2}\partial _{k_1}R^{k_2}_{\ell _2}(x,y){\mathrm {d}}x^{\ell _2}\wedge \cdots \wedge \partial _{\ell _j}\partial _{k_{j-1}}R^{k_j}_{\ell _j}(x,y){\mathrm {d}}x^{\ell _j},\nonumber \\ \end{aligned}$$

where we sum over all indices. Thus permuting everything into the right place we get ${{\,\mathrm{Tr}\,}}(R^j)$, where by abuse of notation we also denote by *R* the appearing curvature. The permutation will give a sign

151 $$\begin{aligned} \prod _{k=1}^{j-1}(-1)^s=(-1)^{\sum _{s=1}^{j-1}s}=(-1)^{j(j-1)/2}. \end{aligned}$$

To get the correct form degree and be consistent with the isomorphism (148), we will need a factor of $u^j$. Indeed, note that $\phi (0,u)=u$, which in fact appears for any $\pi _\hbar ^\varphi$-vertex and hence merging this *j* times we get a factor $u^j$.

One can easily see that the propagator $\mathscr {P}$ will reduce to Kontsevich’s angle propagator $\mathscr {P}(0,z_i)=\frac{1}{2\pi }{\mathrm {d}}\arg (z_i)$ and hence $w_j$ vanishes if *j* is odd. Note that if *j* is even, we have

152 $$\begin{aligned} \int _\mathbb {D}\bigwedge _{k=1}^j\mathscr {P}(0,z_k)=\frac{1}{(2\pi )^j}\int _{\mathbb {D}}\bigwedge _{k=1}^j{\mathrm {d}}\arg (z_k)=\frac{1}{(2\pi )^j}\prod _{k=1}^j\int _{\mathbb {D}}{\mathrm {d}}\arg (z_k)=\prod _{k=1}^j\frac{1}{k}=\frac{1}{j!}.\nonumber \\ \end{aligned}$$

Therefore, as it was computed in [44, 45], we get

153 $$\begin{aligned} w_j=\int _{\mathsf {C}^0_{j+1,0}(\mathbb {D})}\mathscr {P}(z_1,z_2)\wedge \cdots \wedge \mathscr {P}(z_{j},z_1)\wedge \bigwedge _{k=1}^j\mathscr {P}(0,z_k)=-(-1)^{j(j-1)/2}\frac{B_j}{(2j)j!},\nonumber \\ \end{aligned}$$

where $B_j$ are the ***Bernoulli numbers***^14^. Hence we have154Thus we get155Recall from Sect. 2.2 that the ***Connes isomorphism*** $\mathrm {Co}$ is given by

156 $$\begin{aligned} \begin{aligned} \mathrm {Co}:PH_{\bullet }(A)&\longrightarrow H^\bullet (M,\mathbb {R})[u,u^{-1}]\\ [a_0\otimes \cdots \otimes a_m]&\longmapsto \frac{1}{m!}a_0{\mathrm {d}}a_1\wedge \cdots \wedge {\mathrm {d}}a_m. \end{aligned} \end{aligned}$$

Then for any $c\in PC_{\bullet }(A)$ we can see that one gets157In fact, $\mathrm {Co}(c_0)=\mathrm {Ch}(c)$ is the Chern character of the cyclic cycle *c*. Note that if $c=1$ we get $\mathrm {Co}(1)=1$. The map *I* is given by integration  for a chosen volume form $\Omega$ on *M* and thus158$\square$

### Relation to the Nest–Tsygan theorem

Let $M=T^*N$ be the cotangent bundle for a manifold *N* endowed with its canonical symplectic form $\omega$ and consider the constant function 1 on the boundary of the disk. In this setting we get the following theorem.

#### Theorem 8.3

The trace formula (115) satisfies (80).

#### Proof

**Fig. 8:**
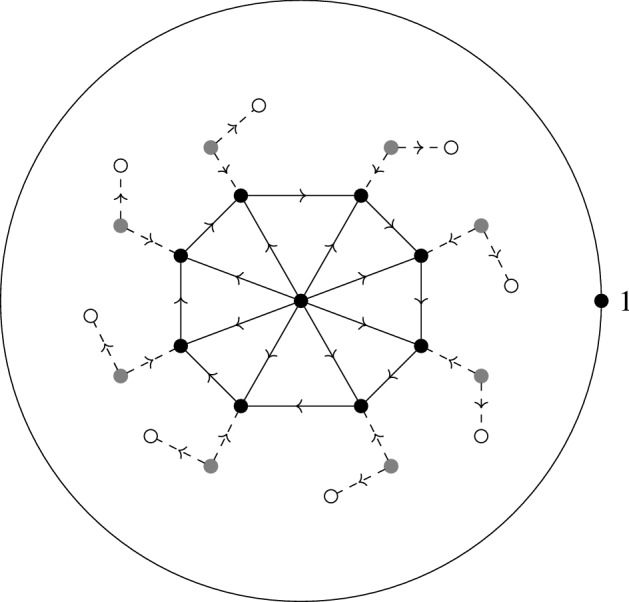
Example of a wheel graph that gives a contribution to the trace formula if we place the constant function 1 on the boundary. The $\pi ^\varphi _\hbar$-vertices are represented by the gray vertices and the *R*-vertices are represented by the black vertices. The picture without the center vertex and the corresponding arrows starting at the center is meant to be before merging. After merging we get the wheel with spokes pointing outwards

**Fig. 9:**
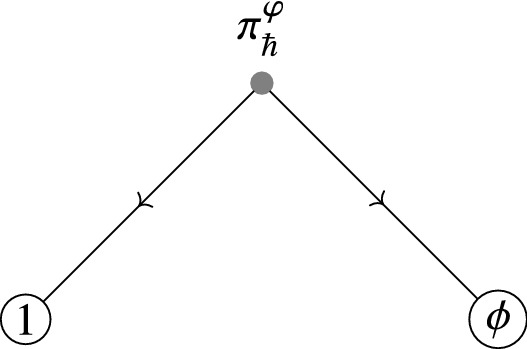
The appearing residual graphs. Here 1 and $\phi$ both are regarded as the generators of the relative equivariant cohomology on the disk $H_{S^1}^\bullet (\mathbb {D},\partial \mathbb {D})$

One can easily check that by Proposition 4.2 and degree reasons the only diagrams contributing within the trace formula are given by wheel-like loops as in Fig. 8, and residual graphs as in Fig. 9. Using the same construction as in Sect. 8.2, we can merge the gray vertices to the center, and obtain wheel graphs which again will give rise to . Recall that , where . Note that we choose $\Omega$ to be the symplectic volume form $\frac{\omega ^d}{d!}$ and, using (89), we can see that if $c=1$, the *u*’s will all cancel each other and thus it will not depend on *u*. Indeed, we have

159 $$\begin{aligned} \exp (\iota _\pi /u)= & {} \sum _{n=0}^\infty \frac{1}{n!}\frac{1}{u^n}(\iota _\pi )^n\nonumber \\= & {} \sum _{n=0}^\infty \frac{\hbar ^n}{n!}\frac{1}{u^n}\prod _{k=1}^n(\mathsf {T}\varphi ^*\omega )^{i_kj_k}\left( \sum _{1\le i_1<j_1<\cdots<i_n<j_n\le 2d}\prod _{k=1}^n\iota _{\partial _{i_k}}\iota _{\partial _{j_k}}\right) ,\qquad \end{aligned}$$

and therefore

160 $$\begin{aligned} \exp (\iota _\pi /u)\frac{\omega ^d}{d!}=\sum _{n= 0}^\infty \sum _{1\le i<j\le 2d} \frac{1}{u^n}\frac{\hbar ^n}{n!d!}\prod _{k=1}^{d-n}(\mathsf {T}\varphi ^*\omega )^{i_kj_k}\bigwedge _{k=1}^d{\mathrm {d}}x^{i_k}\wedge {\mathrm {d}}x^{j_k}. \end{aligned}$$

By degree reasons, the only surviving terms in  are161From the field theoretical construction, it is easy to check that the sum over all residual graphs will exactly give a contribution $\exp \left( \omega _\hbar /\hbar \right)$. Indeed, the integral

162 $$\begin{aligned} \int _\mathbb {D}\phi (z,u)^{s}=\frac{\mathrm {i}}{2\pi }su^{s-1}\int _{\mathbb {D}}(1-\vert z\vert ^2)^{s-1}{\mathrm {d}}z\wedge {\mathrm {d}}{\bar{z}}=u^{s-1},\quad s\ge 1, \end{aligned}$$

and for $s=1$, we get $\int _{\mathbb {D}}\phi =1$. Hence summing over all such graphs we get $\exp (\pi ^\varphi _\hbar )=\exp (\omega _\hbar /\hbar )$. Putting everything together, we have163$\square$

## Relation to the Grady–Li–Li Construction

### The symplectic case

In [33] a similar construction was considered for symplectic manifolds. They formulate a global trace map using the 1-dimensional Chern–Simons theory^15^ within the setting of the BV formalism, by considering solutions of the Quantum Master Equation, and solutions of Fedosov’s equation (22). Moreover, they extend this map to an equivariant one with respect to the $S^1$-action. Let us give some more details for this construction. Let $(M,\omega )$ be a symplectic manifold of dimension 2*d* and consider a symplectic connection $\nabla$ on *TM*. Let164Moreover, define a map165where $\omega ^{ij}$ are the components of $\omega ^{-1}$. Let  denote the complex of differential forms with values in . The symplectic connection can be extended to a map166A degree zero element  is said to satisfy the Quantum Master Equation if

167 $$\begin{aligned} \left( \nabla +\mathrm {i}\hbar \Delta +\frac{\mathrm {i}}{\hbar }{\mathrm {d}}_{TM}F\right) \exp \left( \frac{\mathrm {i}}{\hbar }S\right) =0, \end{aligned}$$

where ${\mathrm {d}}_{TM}$ is the de Rham differential on *TM*, $\Delta :=L_\pi =[{\mathrm {d}}_{TM},\iota _\pi ]$ with $\pi$ the Poisson structure induced by $\omega$ (here *L* denotes the Lie derivative), and *F* is the Weyl curvature tensor given as in (18). In fact  is a BV algebra like as in Appendix 9.3, which is why in [33] they call  the ***BV bundle***. One can show that if (167) is satisfied, the operator $\nabla +\mathrm {i}\hbar +\{S,\}_\Delta$ is a differential on . Here $\{,\}_\Delta$ denotes the odd Poisson bracket defined by $\Delta$. For a solution *S* of (167), we define the ***twisted integration map***168In fact, one can show that169is a cochain map and hence, by composition, we have a map170Fix a solution $\gamma$ of (22). Then one can construct a nilpotent^16^ solution $\gamma _\infty$ of (167) as an effective action

171 $$\begin{aligned} \gamma _\infty :=-\mathrm {i}\hbar \log \sum _{\Gamma \in \mathcal {G}^0}\frac{\hbar ^{\ell (\Gamma )}}{\vert \mathrm {Aut}(\Gamma )\vert }\int _{\mathsf {C}_\Gamma (S^1)}\omega _\Gamma (\gamma ,\mathscr {P}_{S^1}), \end{aligned}$$

where $\mathcal {G}^0$ denotes the set of all connected graphs, $\ell (\Gamma )$ denotes the number of loops of $\Gamma$, $\mathrm {Aut}(\Gamma )$ denotes the automorphism group of $\Gamma$, and $\omega _\Gamma (\gamma ,\mathscr {P}_{S^1})$ a differential form depending on a chosen propagator $\mathscr {P}_{S^1}$ on $S^1$ and $\gamma$.

Define a map172which represents a ***factorization map*** from local observables on the interval to global observables on $S^1$. The trace map in this setting is defined by

173 $$\begin{aligned} \begin{aligned} {{\,\mathrm{Tr}\,}}_\infty :C^\infty (M)[\![ \hbar ]\!]&\longrightarrow \mathbb {R}(\!( \hbar )\!)\\ f&\longmapsto {{\,\mathrm{Tr}\,}}_\infty (f):=\int _M\int _{\gamma _\infty }[\sigma ^{-1}(f)]_\infty , \end{aligned} \end{aligned}$$

where $\sigma$ is the symbol map (23).

For the equivariant formulation, extend the map $\sigma$ to the BV bundle174by sending $y^i,{\mathrm {d}}y^i\mapsto 0$, and define the $S^1$-equivariantly extended complexes

175 $$\begin{aligned} \Omega ^\bullet (M,\mathcal {W})^{S^1}:=\left( \Omega ^\bullet (M,\mathcal {W})[u,u^{-1},{\mathrm {d}}t],\nabla +\frac{1}{\hbar }[\gamma ,]_\star -u\iota _{\frac{{\mathrm {d}}}{{\mathrm {d}}t}}\right) , \end{aligned}$$

where *t* is the coordinate on $S^1$, and176Moreover, one can extend the map $[]_\infty$ to an equivariant version177and show that it still remains a cochain map for the equivariant differentials. Furthermore, one also defines an ***equivariant twisted integration map***178

#### Remark 9.1

In fact one can show that (178) extends (168) as179

Again, one can show that (178) remains a cochain map with respect to the extended complexes, and in particular the composition

180 $$\begin{aligned} \int _{\gamma _\infty }^{S^1}[]_\infty ^{S^1}:\Omega ^\bullet (M,\mathcal {W})^{S^1}\longrightarrow \Omega ^\bullet (M,\mathbb {R})(\!( \hbar )\!)[u,u^{-1}] \end{aligned}$$

is a cochain map. The $S^1$-equivariant trace map is then defined by

181 $$\begin{aligned} \begin{aligned} {{\,\mathrm{Tr}\,}}_\infty ^{S^1}:\Omega ^\bullet (M,\mathcal {W})^{S^1}&\longrightarrow \mathbb {R}(\!( \hbar )\!)[u,u^{-1}]\\ f&\longmapsto {{\,\mathrm{Tr}\,}}_\infty ^{S^1}(f)=\int _M\int _{\gamma _\infty }^{S^1}[f]_\infty ^{S^1}. \end{aligned} \end{aligned}$$

Moreover, the relation to (173) is

182 $$\begin{aligned} {{\,\mathrm{Tr}\,}}_\infty (f)={{\,\mathrm{Tr}\,}}_\infty ^{S^1}({\mathrm {d}}t\sigma ^{-1}(f)). \end{aligned}$$

### Feynman graphs for cotangent targets

Consider the case of the Poisson Sigma Model with target a cotangent bundle $M=T^*N$ for some manifold *N*. Then by Proposition 4.2 and Lemma 7.6 the graphs will reduce to a certain class of graphs. We have two different bulk vertices. There are vertices labeled by $\pi _\hbar ^\varphi$ and vertices labeled by *R*. The $\pi _\hbar ^\varphi$-vertices emanate two arrows, representing $\bar{q}$- and ${\bar{p}}$-derivatives as in Sect. 4.3, and there are no arrows arriving at them, since the Poisson structure is constant. The *R*-vertices emanate one arrow and there can be an arbitrary amount of arrows representing ${\bar{q}}$-derivatives arriving at them, but by Proposition 4.2 we can only have at most one arrow representing a ${\bar{p}}$-derivative arriving. We also consider vertices on the boundary representing solutions $\gamma$ of (42). For each of them there are no arrows emanating and arbitrarily many arriving.

**Fig. 10:**
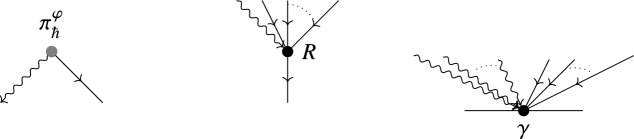
The interaction vertices appearing in the cotangent case. The straight arrows represent a ${\bar{q}}$-derivative and the wavy arrows represent a ${\bar{p}}$-derivative. There are no incoming arrows at the $\pi _\hbar ^\varphi$-vertices and exactly two emanating arrows. There are arbitrarily many incoming arrows representing the ${\bar{q}}$-derviatives for an *R*-vertex, but at most one arrow representing a ${\bar{p}}$-derivative and exactly one arrow emanating. For the $\gamma$-vertices we have arbitrarily many incoming $\bar{q}$- and ${\bar{p}}$-derivatives and no emanating arrows

#### Example 9.2

**Fig. 11:**
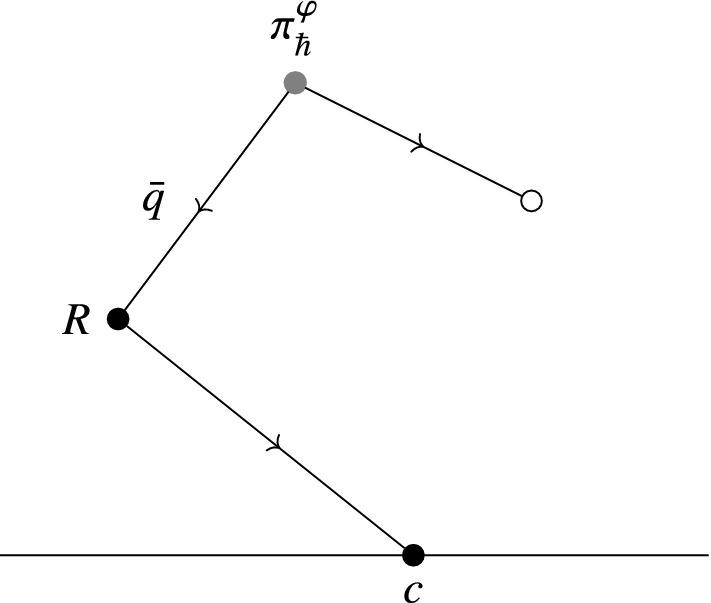
Example of a graph contributing to the trace formula for a cotangent target. Note there is no ${\bar{p}}$-derivative for the *R*-vertex. Moreover, one can check that it provides a correct degree count. Indeed, the amount of black vertices in the bulk is given by 2, hence $\dim \mathsf {C}^0_\Gamma (\mathbb {D})=4$ and the form degree of $\omega _\Gamma$ is given by $\vert R\vert +1+2=4$

**Fig. 12:**
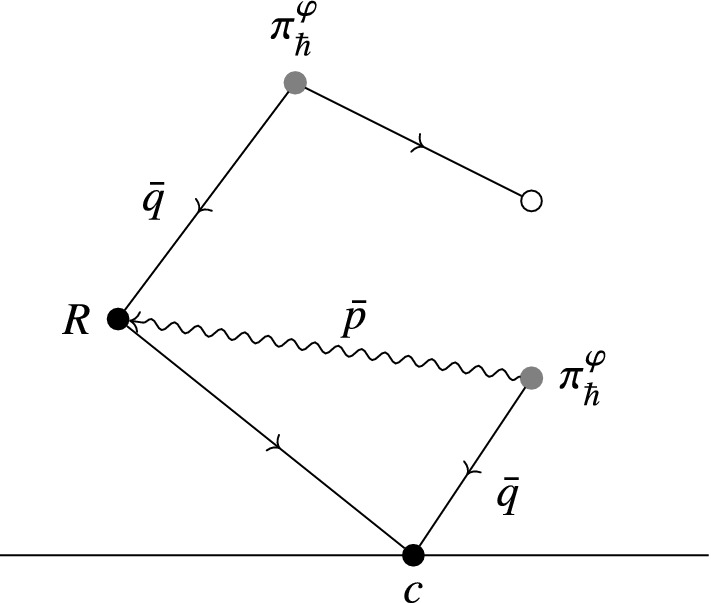
Example of a graph contributing to the trace formula for a cotangent target. Note there is only one ${\bar{p}}$-derivative for the *R*-vertex. Moreover, one can check that it provides a correct degree count. Indeed, the amount of black vertices in the bulk is given by 3, hence $\dim \mathsf {C}^0_\Gamma (\mathbb {D})=6$ and the form degree of $\omega _\Gamma$ is given by $\vert R\vert +\vert \pi ^\varphi _\hbar \vert +1+2=6$

Examples of graphs appearing for cotangent targets are given in Figs. 11 and 12.

### Reduction of the trace formula for cotangent targets

#### Proposition 9.3

The trace map for the globalized Poisson Sigma Model with cotangent target reduces to the trace map (181).

#### Proof

Consider the Poisson Sigma Model with target a cotangent bundle $M=T^*N$ for some manifold *N* such that $\dim M=2d$. The Poisson structure is then induced by the canonical symplectic form $\omega$ on *M*. Note first that (115) can be written as

183 $$\begin{aligned} {{\,\mathrm{Tr}\,}}^\mathcal {V}(f)=\int _M\rho (\mathsf {T}\varphi ^*f)\vert _{y=0}\exp (\mathsf {T}\varphi ^*h\vert _{y=0})\Omega +O(\hbar )=\int _Mf\exp (h)\Omega +O(\hbar ),\nonumber \\ \end{aligned}$$

where *h* was the Hamiltonian function for $\pi$ such that ${{\,\mathrm{\text {div}}\,}}_\Omega \pi -[h,\pi ]=0$. Indeed, by considering the Feynman graph expansion of $\mathcal {V}_n^{\pi ^\varphi _\hbar }$, we get that

184 $$\begin{aligned} {{\,\mathrm{Tr}\,}}^\mathcal {V}(f)=\int _M\sum _{n=0}^\infty \frac{\hbar ^n}{n!}P_n(\mathsf {T}\varphi ^*\pi ,\mathsf {T}\varphi ^*h,\rho (\mathsf {T}\varphi ^*f))\exp (\mathsf {T}\varphi ^*h)\Omega , \end{aligned}$$

where $P_n$ are differential polynomials in $\mathsf {T}\varphi ^*\pi ,\mathsf {T}\varphi ^*h$, and $\rho (\mathsf {T}\varphi ^*f)$. Now, considering cotangent targets and choosing $\Omega$ to be the symplectic volume $\frac{\omega ^d}{d!}$, we can see that the leading order $\hbar$ term of ${{\,\mathrm{Tr}\,}}(f)$ is given by

185 $$\begin{aligned} \int _Mf\frac{\omega ^d}{d!}. \end{aligned}$$

Since $\pi$ is constant, we get that ${{\,\mathrm{\text {div}}\,}}_\Omega \pi =0$ and hence $[h,\pi ]=0$, which implies that *h* is constant e.g., $h=0$. This is also compatible with the Nest–Tsygan theorem. One can compute186Note that we have used . Using the Feynman graphs for the corresponding effective theory together with the fact that, for a solution $\gamma$ of (22), the leading $\hbar$ term of ${\mathrm {d}}_{TM}\gamma$ is given by $\omega _{ij}{\mathrm {d}}y^i\wedge {\mathrm {d}}x^j$, it can be seen that the leading $\hbar$ term of (181) is given by

187 $$\begin{aligned} \int _M\int _{Ber}f\exp (\omega _{ij}{\mathrm {d}}y^i\wedge {\mathrm {d}}x^j/\hbar )=\frac{(-1)^d}{\hbar ^d}\int _Mf\frac{\omega ^d}{d!}. \end{aligned}$$

Here we use that the map $\int _{Ber}$ will give rise to the symplectic volume form $\frac{\omega ^d}{d!}$ on *M*. Therefore the $\hbar$ leading terms coincide. Moreover, in [33] they also show how the trace (173) is compatible with the Nest–Tsygan theorem. Note that the morphism $\mathcal {V}^{\pi ^\varphi _\hbar }$, which is given as the expectation value of the Fedosov-type formal global action, gives rise to the twisted integration map, where the effective action is indeed given in terms of a solution $\gamma$ of (22) since (43) is reduced to (22) for the symplectic case as explained in Sect. 4.5. This action functional corresponds to $\gamma _\infty$, which can bee seen by using the corresponding Feynman rules on $S^1$. Moreover, the Grothedieck connection gives rise to the globalization map $[]^{S^1}_\infty$ for observables and the quantization map $\rho$ reduces to the inverse of the symbol map $\sigma$. $\quad \square$

## Appendix A. BV Algebras and Relation to Field Theory

We want to recall some notions on BV algebras as in [31], and how it is related to the original gauge formalism developed by Batalin and Vilkovisky within quantum field theory.

### A.1. Braid algebras

Let us first recall what a braid algebra is. A braid algebra *B* is a commutative DG algebra endowed with a Lie bracket $[,]$ of degree $+1$ satisfying the Poisson relations

188 $$\begin{aligned}{}[a,bc]=[a,b]c+(-1)^{\vert a\vert (\vert b\vert -1)}b[a,c],\qquad \forall a,b,c\in B \end{aligned}$$

An identity element in *B* is an element $\varvec{1}$ of degree 0 such that it is an identity for the product and $[\varvec{1},]=0$.

### A.2. BV algebras

A BV algebra *A* is a commutative DG algebra endowed with an operator $\Delta :A_\bullet \longrightarrow A_{\bullet +1}$ such that $\Delta ^2=0$ and

189 $$\begin{aligned} \begin{aligned} \Delta (abc)&=\Delta (ab)c+(-1)^{\vert a\vert }a\Delta (bc)+(-1)^{(\vert a\vert -1)\vert b\vert }b\Delta (ac)\\&\quad -\Delta (a)bc-(-1)^{\vert a\vert }a\Delta (b)c-(-1)^{\vert a\vert +\vert b\vert }ab\Delta (c),\qquad \forall a,b,c\in A. \end{aligned}\nonumber \\ \end{aligned}$$

An identity in *A* is an element $\varvec{1}$ of degree 0 such that it is an identity for the product and $\Delta (\varvec{1})=0$. One can show that a BV algebra is in fact a special type of a braid algebra. More precisely, a BV algebra is a braid algebra endowed with an operator $\Delta :A_\bullet \longrightarrow A_{\bullet +1}$ such that $\Delta ^2=0$ and such that the bracket and $\Delta$ are related by

190 $$\begin{aligned} {[}a,b]=(-1)^{\vert a\vert }\Delta (ab)-(-1)^{\vert a\vert }\Delta (a)b-a\Delta (b),\qquad \forall a,b\in A. \end{aligned}$$

Moreover, in a BV algebra we have

191 $$\begin{aligned} \Delta ([a,b])=[\Delta (a),b]+(-1)^{\vert a\vert -1}[a,\Delta (b)],\qquad \forall a,b\in A. \end{aligned}$$

### A.3. Connection to field theory

We would like to explain the name “BV” algebra. This comes from the approach to deal with gauge theories in quantum field theory developed by Batalin–Vilkovisky in the setting of odd symplectic (super)manifolds. Let $(\mathcal {F},\omega )$ be an odd symplectic (super)manifold. In physics, $\mathcal {F}$ is called the space of fields. Let $f\in C^\infty (\mathcal {F})$ and consider its Hamiltonian vector field $X_f$. One can check that $C^\infty (\mathcal {F})$ endowed with the Poisson bracket

192 $$\begin{aligned} \{f,g\}:=(-1)^{\vert f\vert -1}X_f(g) \end{aligned}$$

is a braid algebra. Let $\mu \in \Gamma (Ber(\mathcal {F}))$ be a nowhere-vanishing section of the Berezinian bundle of $\mathcal {F}$. This represents a density which is characterized by the integration map $\int :\Gamma _c(\mathcal {F},Ber(\mathcal {F}))\longrightarrow \mathbb {R}$. Hence $\mu$ induces an integration map on functions with compact support

193 $$\begin{aligned} C^\infty (\mathcal {F})\ni f\longmapsto \int _{\mathcal {L}\subset \mathcal {F}} f\mu ^{1/2}, \end{aligned}$$

for some Lagrangian submanifold $\mathcal {L}\subset \mathcal {F}$, where the integral exists. Then one can define a divergence operator ${{\,\mathrm{\text {div}}\,}}_\mu X$ by

194 $$\begin{aligned} \int _\mathcal {F}({{\,\mathrm{\text {div}}\,}}_\mu X)f\mu =-\int _\mathcal {F}X(f)\mu . \end{aligned}$$

#### Lemma A.1

For a vector field *X* let $X^*=-X-{{\,\mathrm{\text {div}}\,}}_\mu X$. Then

195 $$\begin{aligned} \int _\mathcal {F}fX(g)\mu =(-1)^{\vert f\vert \vert X\vert }\int _\mathcal {F}X^*(f)g\mu . \end{aligned}$$

Moreover, ${{\,\mathrm{\text {div}}\,}}_\mu (fX)=f{{\,\mathrm{\text {div}}\,}}_\mu X-(-1)^{\vert f\vert \vert X\vert }X(f)$ and if $S\in C^\infty (\mathcal {F})$ is an even function, then ${{\,\mathrm{\text {div}}\,}}_{\exp (S)\mu }X={{\,\mathrm{\text {div}}\,}}_\mu X+X(S)$.

One can then define $\Delta$ to be the odd operator on $C^\infty (\mathcal {F})$ given by

196 $$\begin{aligned} \Delta (f)={{\,\mathrm{\text {div}}\,}}_\mu X_f. \end{aligned}$$

A BV (super)manifold $(\mathcal {F},\omega ,\mu )$ is then an odd symplectic (super)manifold with Berezinian $\mu$ such that $\Delta ^2=0$.

#### Proposition A.2

Let $(\mathcal {F},\omega ,\mu )$ be a BV (super)manifold.

1. The algebra $(C^\infty (\mathcal {F}),\{,\},\Delta )$ is a BV algebra, where $\Delta$ is given as in (196) and $\{,\}$ is the odd Poisson bracket coming from the odd symplectic form $\omega$ as in (192).
2. The Hamiltonian vector field associated to some $f\in C^\infty (\mathcal {F})$ is given by the formula $X_f=-[\Delta ,f]+\Delta (f)$, where $[,]$ denotes the commutator of operators.
3. If $S\in C^\infty (\mathcal {F})$ and $\Delta _S$ is the operator associated to the Berezinian $\exp (S)\mu$, then $\Delta _S=\Delta -X_S$ and $\Delta _S^2=X_{\Delta (S)+\frac{1}{2}\{S,S\}}$.

Note that point (3) is exactly the case that we have in quantum field theory. Moreover, if

197 $$\begin{aligned} \Delta (S)+\frac{1}{2}\{S,S\}=0, \end{aligned}$$

we get that $\Delta _S^2=0$, which ensures a BV algebra structure. In physics, the function *S* is called the action and Equation (197) is usually called the Quantum Master Equation^17^.

## Footnotes

1 Let $A=C^\infty (M)$ for some Poisson manifold *M*. For a deformed algebra $(A[\![\hbar ]\!],\star )$, closedness means that the integration of elements of the deformed algebra is a trace with respect to $\star$. In particular, a star product is closed if and only if $\int _M (f\star g)\Omega =\int _M(g\star f)\Omega$ for any $f,g\in A$, where $\Omega$ is a volume form on *M*.

2 We will use the Einstein summation convention.

3 Typically, one only speaks of a Weyl bundle if the underlying manifold *M* is symplectic. However, the construction is general for any manifold *M*.

4 Recall that for a function *f* one can construct a unique vector field $X_f$, such that $\iota _{X_f}\omega =-{\mathrm {d}}f$; the vector field $X_f$ is called the Hamiltonian vector field of *f* and *f* is called the Hamiltonian function of $X_f$.

5 In fact, the Poisson Sigma Model is a nontrivial example of a gauge theory where the BV formalism is actually needed for quantization, showing the importance of the formalism.

6 One should be aware that this construction is only formal, since the discussion in Appendix A only applies to finite-dimensional manifolds.

7 Note that we are secretly using a formal exponential map $\varphi$. In particular, $\xi _k$ should be $\varphi ^*\xi _k$.

8 The Poisson homology $HP_\bullet (M)$ of a Poisson manifold $(M,\pi )$ is given by the homology of the complex $(\mathcal {T}_{poly}^\bullet (M),[\pi ,])$, where $[\pi ,]$ is the Poisson differential of degree $-1$ (note that we have taken the opposite grading).

9 Note that we write $\xi _1\cdots \xi _n$ for the symmetric tensor product $\xi _1\otimes \cdots \otimes \xi _n$ of multivector fields $\xi _1,\ldots ,\xi _n\in \mathcal {T}_{poly}^\bullet (M)$.

10 Note that this does not depend on *t*, since we fix it on the boundary.

11 This argument follows from Stokes’ theorem and the “bubbling” concept of the Deligne–Mumford compactification on the disk.

12 In addition to the rules below we will also consider it modulo graph isomorphisms which respect the partition and the orderings. The set $\mathcal {G}_{\mathbf {k},m}$ is in principle given by the set of equivalence classes.

13 We use the same notation as in Lemma 7.4.

14 Note that this makes indeed sense since the $B_j$ is zero for odd $j\not =1$. The case of $j=1$ is not relevant since *j* has to be at least two.

15 This is simply the case of topological quantum mechanics with action $S=\int _{S^1} p{\mathrm {d}}q$.

16 i.e. there is some $N\gg 0$ such that $\gamma _\infty ^N=0$, which is in fact true since the exponential map will terminate for some power.
